# Integration of microarray analysis into the clinical diagnosis of hematological malignancies: How much can we improve cytogenetic testing?

**DOI:** 10.18632/oncotarget.4586

**Published:** 2015-07-31

**Authors:** Jess F. Peterson, Nidhi Aggarwal, Clayton A. Smith, Susanne M. Gollin, Urvashi Surti, Aleksandar Rajkovic, Steven H. Swerdlow, Svetlana A. Yatsenko

**Affiliations:** ^1^ Pittsburgh Cytogenetics Laboratory, Center for Medical Genetics and Genomics, Magee-Womens Hospital of UPMC, Pittsburgh, PA, USA; ^2^ Department of Human Genetics, University of Pittsburgh Graduate School of Public Health, Pittsburgh, PA, USA; ^3^ Department of Pathology, University of Pittsburgh School of Medicine, Pittsburgh, PA, USA; ^4^ Department of Medicine, Division of Hematology, University of Colorado, Denver, CO, USA; ^5^ Department of Obstetrics, Gynecology and Reproductive Sciences, University of Pittsburgh School of Medicine, Pittsburgh, PA, USA; ^6^ Department of Pathology, Medical College of Wisconsin, Milwaukee, WI, USA

**Keywords:** Chromosome Section, hematological malignancies, microarray, diagnosis, array CGH

## Abstract

**Purpose:**

To evaluate the clinical utility, diagnostic yield and rationale of integrating microarray analysis in the clinical diagnosis of hematological malignancies in comparison with classical chromosome karyotyping/fluorescence *in situ* hybridization (FISH).

**Methods:**

G-banded chromosome analysis, FISH and microarray studies using customized CGH and CGH+SNP designs were performed on 27 samples from patients with hematological malignancies. A comprehensive comparison of the results obtained by three methods was conducted to evaluate benefits and limitations of these techniques for clinical diagnosis.

**Results:**

Overall, 89.7% of chromosomal abnormalities identified by karyotyping/FISH studies were also detectable by microarray. Among 183 acquired copy number alterations (CNAs) identified by microarray, 94 were additional findings revealed in 14 cases (52%), and at least 30% of CNAs were in genomic regions of diagnostic/prognostic significance. Approximately 30% of novel alterations detected by microarray were >20 Mb in size. Balanced abnormalities were not detected by microarray; however, of the 19 apparently “balanced” rearrangements, 55% (6/11) of recurrent and 13% (1/8) of non-recurrent translocations had alterations at the breakpoints discovered by microarray.

**Conclusion:**

Microarray technology enables accurate, cost-effective and time-efficient whole-genome analysis at a resolution significantly higher than that of conventional karyotyping and FISH. Array-CGH showed advantage in identification of cryptic imbalances and detection of clonal aberrations in population of non-dividing cancer cells and samples with poor chromosome morphology. The integration of microarray analysis into the cytogenetic diagnosis of hematologic malignancies has the potential to improve patient management by providing clinicians with additional disease specific and potentially clinically actionable genomic alterations.

## INTRODUCTION

Classical cytogenetic analysis (G-banding) plays a critical role in the diagnosis, prognosis and treatment planning of hematologic malignancies [1–16], and enables the detection of large genomic alterations, including aneusomies, balanced and unbalanced chromosomal rearrangements of at least 10–20 Mb in size, and mosaicism. However, the necessity for dividing cells, sometimes poor chromosome morphology, and the relatively low-resolution of G-banding are limiting factors for a complete and accurate chromosome study.

Fluorescence *in situ* hybridization (FISH) has proven invaluable as an ancillary technique for the identification of clinically significant chromosomal aberrations, as FISH can be performed on metaphase or non-dividing interphase cells, and can detect genomic abnormalities with a resolution from 150 to 900 kb, depending on the probe size. In hematologic malignancies, FISH enables the rapid detection of: 1) fusion genes amenable to targeted therapy (e.g., *PML*-*RARA, ABL1-BCR*); 2) other recurrent cytogenetic abnormalities of diagnostic or prognostic importance that may be present in quiescent cells, including both chromosomal rearrangements and copy number abnormalities; 3) submicroscopic copy number changes of clinical significance, such as deletions involving *TP53* and *ATM*; 4) relatively low-level mosaicism for clonal aberrations by evaluation of a large population of interphase cells; and 5) may facilitate the characterization of complex chromosome rearrangements identified by G-banded analysis. Most laboratories use a set of commercially available, single-locus probes to detect genetic changes in specific, targeted genomic regions that are diagnostic or prognostic for a certain type of hematologic malignancy, such as FISH panels for acute myeloid leukemia (AML) or acute lymphoblastic leukemia (ALL). Despite numerous advantages, FISH does not provide genome-wide analyses and must be targeted to selected genomic regions believed to be of interest in a given case. FISH may yield false negative results in cases where genomic imbalances are smaller than the size of a FISH probe or chromosomal rearrangements are complex; or false positive results when two fluorescent signals co-localize due to viewing a three-dimensional nucleus in two dimensions.

The advent of high-resolution genome arrays, including comparative genomic hybridization (CGH) and single nucleotide polymorphism (SNP), has enabled the detection of submicroscopic copy number variations of clinical significance, in addition to the majority of abnormalities identifiable by G-banding and FISH [17–22]. Microarray analysis eliminates the need for dividing cells and can be performed on direct (uncultured) specimens to provide a more accurate assessment of abnormalities and tumor burden. SNP arrays also can detect genomic regions with loss of heterozygosity (LOH), a clinically significant aberration associated with hematologic and other malignancies [17, 18, 22–24]. Array-CGH cannot detect balanced rearrangements or genomic imbalances present in less than 10–20% of cells. Furthermore, the precise physical location of genomic gains cannot be determined by microarray analysis and requires G-banding or FISH for further characterization.

In the past decade, advances in genomic technologies, such as high-throughput sequencing and whole genome microarray studies have provided a wealth of insights into mechanisms of cancer development and evolution. Molecular pathways have been identified that drive cancer cell transformation and progression, convey tumor clinical behavior and response to therapy. As a result, an international working group reported a number of recommendations giving special attention to the detection of disease-associated cytogenetic and molecular defects, prognostic genetic markers, and novel targeted therapeutic options for hematologic malignancies [4–9].

The 2008 WHO classification of hematologic malignancies incorporates cytogenetic abnormalities that are mainly well-recognized using classical cytogenetic studies with limited categories requiring FISH studies or mutational analyses [1]. To date, for example, genetic lesions in at least 21 genes have been implicated in pathogenesis of myelodysplastic syndrome (MDS) [4]. The International Prognositic Scoring System (IPSS) and the National Comprehensive Cancer Network (NCCN; www.nccn.org) guidelines recognize five prognostic categories in MDS that include evaluation for inv(3)/t(3)/del(3q), del(5q), −7/del(7q), +8, del(12p), +19, and del(20q). In addition, FISH analyses for *RUNX1-RUNX1T1*, *CBFB-MYH11*, and *PML*-*RARA* may be necessary to exclude AML. Further genetic screening for submicroscopic deletions and duplications, along with point mutations is recommended in patients with familial MDS/AML. Although large genomic alterations are readily detectable by classical cytogenetic techniques, the low-resolution of G-banding is insufficient to detect submicroscopic aberrations. Thus, the burden falls upon FISH, and the continued expansion of FISH panels is infeasible due to prolonged turn-around time, cost, and the inability to target all genomic regions of clinical interest. Therefore, it is critical for cytogenetics laboratories to adopt technology that will provide clinicians with results as quickly and efficiently as possible.

To date, multiple studies have shown the value of microarray analysis in the diagnosis of hematologic malignancies [17–24]. Here we present the results of a comprehensive comparison between classical chromosome karyotyping (G-banding), FISH, and microarray studies, and demonstrate the benefits, limitations and rationale of microarray analysis in the clinical diagnosis of hematologic malignancies.

## RESULTS

Twenty-seven specimens were analyzed for DNA copy number variations (CNVs) using either the CGH or CGH+SNP microarray design (GEO accession no. GSE66960, http://www.ncbi.nlm.nih.gov/geo/query/acc.cgi?token=svsnkiyatnmbnyz&acc=GSE66960) and compared with results of clinical G-banding and/or FISH studies (Supplementary Table S1 and S2).

**Figure 1:**
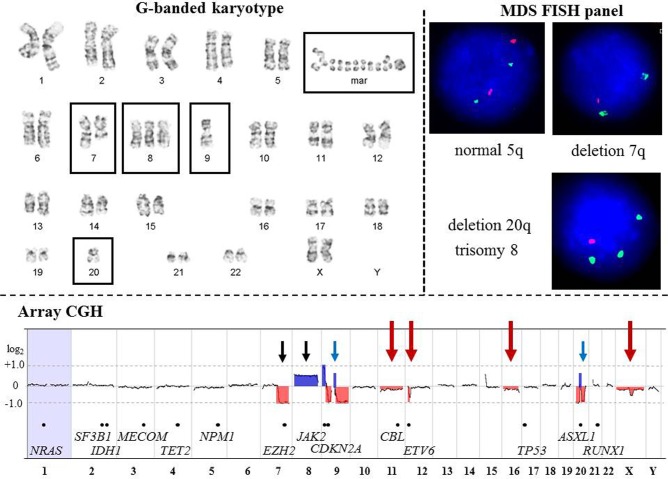
Comparison of G-banding, FISH, and array-CGH results from a patient (CP-13) with Myelodysplastic Syndrome **G-banded karyotype.** Karyotype at the 400-band resolution showing an abnormal clone with five abnormalities (boxed): deletion of the long arm of chromosome 7; trisomy 8; monosomies 9 and 20; 1 to 4 marker chromosomes; and 4 to 27 double minutes. According to WHO classification (2008), 50% of MDS cases have gross cytogenetic aberrations at diagnosis including deletion 5q, deletion 7q or monosomy 7, trisomy 8, and deletion 20q. Approximately 30% of MDS patients will acquire additional genomic aberrations and develop acute myeloid leukemia (AML). To date, mutations, fusion transcripts, deletions, duplications and amplifications in at least 21 genes are associated with transformation of MDS into AML. Classical cytogenetic analysis enables whole-genome evaluation of malignant clones; however, cryptic chromosomal abnormalities will be missed due to the low-resolution of this technique. **MDS FISH panel.** The MDS FISH panel using single-locus probes consists of three hybridization experiments to detect four common genomic imbalances [del(5q), del(7q), trisomy 8, and del(20q)] in interphase nuclei. FISH is a targeted approach to identify abnormalities in specific regions of the genome. Therefore, the extent and complexity of chromosomal deletion/duplication, as well as aberrations involving the other chromosomal regions will remain undetected. In MDS patients with a high-risk for transformation into AML, additional FISH studies or an AML FISH panel are frequently requested. **Array-CGH.** High-resolution whole-genome profiling by microarray identified multiple genomic imbalances. Microarray probes are arranged according to their physical map locations on each chromosome from the distal p-arm (on the left) to the distal q-arm (on the right). Chromosomes are plotted in a horizontal fashion and are listed at the bottom. An average logarithmic ratio (log_2_) is displayed for all oligonucleotide probes. Probes with a log_2_ ratio clustered around zero indicate DNA segments with normal copy numbers. A positive log_2_ ratio (above zero) indicates a gain (extra copy) of the chromosomal region, while intervals with a negative log_2_ ratio (below zero) represent loss of DNA copy number. Deletion 7q and trisomy 8 are concordant (black arrows) with the results of G-banding and FISH analyses, while array-CGH revealed a loss of most of chromosomes 9 and 20 (blue arrows). Microarray analysis also detected multiple 9p, 9q, and 20q segments with a gain or no change in DNA copy number, consistent with a complex rearrangement (partially concordant results with G-banded studies). In addition, array-CGH revealed abnormalities missed by G-banding or FISH (red arrows), including: mosaic monosomies 11 and 16; a deletion in the short arm of chromosome 12 (12p13.2 region); mosaic loss of one X chromosome, and a deletion of the long arm of the chromosome X (Supplementary Table S2). Genes implicated in MDS and AML transformation is indicated by black dots along the array-CGH plot.

**Figure 2:**
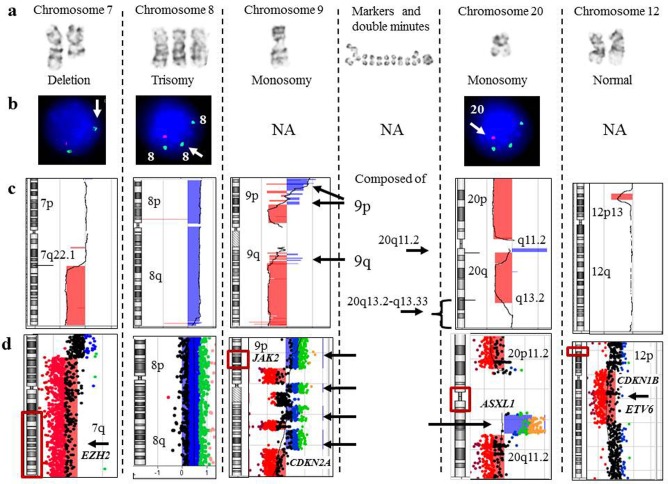
Focused evaluation of aberrations detected by G-banding, FISH, and array-CGH from a patient (CP-13) with myelodysplastic syndrome **a.** Aberrant chromosomes determined by G-banding karyotyping. **b.** The results of MDS FISH panel studies. **c.** A chromosome view of array-CGH analysis. Regions with a DNA copy number loss are indicated in red, and intervals with gains are highlighted in blue. **d.** A magnified view of the rearranged chromosomal regions. On the left, an idiogram with a red box showing a region of interest. On the right, array-CGH plot depicting rearrangement. There was complete concordance among all methodologies for deletion 7q and trisomy 8. For monosomies 9 and 20, G-banding was partially concordant with array-CGH analysis. Alternating regions of gain and loss spanning chromosome 9 and amplification of a proximal region of 20q most likely account for the marker chromosomes and double minutes identified by G-banding. The MDS FISH panel does not target chromosome 9 (NA, FISH is not applicable), and because a single probe for 20q is utilized, the amplified 20q region was not detected. Lastly, microarray identified a deletion, spanning the short arm of chromosome 12, that included the *ETV6* gene, which has been implicated in MDS transformation to AML.

For example, we compared G-banding, FISH (MDS panel), and array-CGH results in a patient with a provisional diagnosis of MDS (Figure 1). Array-CGH detected specific MDS-associated aberrations [del (7q), del(20q), and +8], additional genomic changes consistent with MDS evolving into AML, and complex chromosome alterations suggestive of chromothripsis (Figure 2). In contrast to the results of G-banding and FISH studies, microarray analysis provided definitive genomic alterations of diagnostic and prognostic significance and eliminated the need for additional FISH studies.

### Completely concordant results between G-banding/FISH and microarray analyses

**Figure 3:**
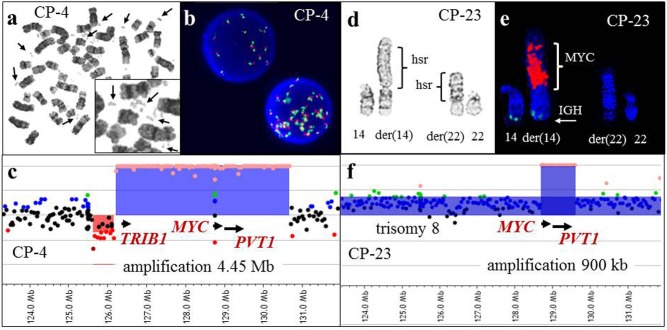
G-banding, FISH, and array-CGH analysis in two cases with 8q24 amplification **a.** A metaphase spread from CP-4 showing double minutes (arrows) detected by G-banded classical cytogenetic analysis. **b.** Interphase FISH analysis demonstrated 8q24 amplification using the *MYC* break-apart probe (Vysis). **c.** A magnified view of the array-CGH plot. Each dot represents an oligonucleotide probe. The black dots indicate probes without change in copy number and red dots indicate a single copy loss. Chromosomal gain suggestive of one, two or multiple extra copies is indicated by blue, green and orange dots, respectively. Abnormal regions with at least five consecutive probes deviating from the normal log_2_ ratio are highlighted in red (deletions) or in blue (duplications, triplications, or amplifications). Array-CGH analysis demonstrated amplification (orange dots) of 8q24.13-q24.21, which includes the *TRIB1*, *MYC* and *PVT1* genes (blue shaded area). **d.** A partial karyotype from CP-23 showing the der(14) and der(22) chromosomes, both with homogenously staining regions (hsr). **e.** Partial karyotype of the same metaphase cell subjected to FISH analysis utilizing the *IGH* (green)/*MYC* (red) dual fusion translocation probe set. The red signal hybridized to the hsr region on the der(14) chromosome, consistent with *MYC* amplification. **f.** Array-CGH analysis demonstrated gain of whole chromosome 8-specific probes (blue dots), consistent with trisomy 8, and amplification of 8q24.21 (orange dots), spanning the *MYC* gene. FISH analysis for *MYC* amplification was initiated due to the microarray findings, and aided in the interpretation of the classical karyotype.

**Figure 4:**
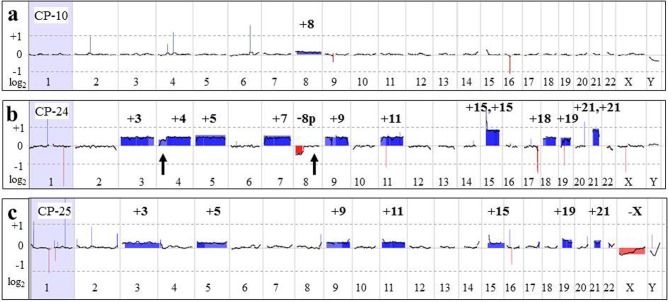
Large chromosomal aberrations detected by aCGH analysis **a.** Whole genome microarray plot from CP-10. An increased log_2_ average was detected for all chromosome 8-specific probes, indicating low-level mosaicism (~7% based on FISH results) for trisomy 8, consistent with results of G-banded karyotyping and FISH studies. **b.** The array-CGH plot from CP-24 shows gain of all probes specific for chromosomes 3, 4, 5, 7, 9, 11, 15, 18, 19, and 21 (blue-shaded areas), and loss of 8p-specific probes (red-shaded). Please note the average log_2_ = 0.95, indicative of tetrasomy for chromosomes 15 and 21. Microarray and G-banding results were discordant for two segments: distal 4p and 8q (arrows), suggestive of mosaic monosomy 4p in addition to trisomy 4 and an 8p deletion versus monosomy 8. Retrospective review of G-banded cells revealed one metaphase cell with poor chromosome morphology, showing 8q attached to 4p, accounting for the discrepancy. Suboptimal chromosome morphology and an insufficient number of metaphase cells precluded classical cytogenetic recognition of this abnormality associated with clonal evolution, whereas it was detected by array-CGH. **c.** The microarray plot from CP-25, showing gain of chromosomes 3, 5, 9, 11, 15, 19, and 21 and loss of the X chromosome. FISH analysis for 11q- and 5q-specific probes showed an extra signal in ~7% of cells.

Findings of G-banding/FISH and microarray analyses were concordant in 5 of 27 cases (18.5%) (CP-4, CP-10, CP-12, CP-16, and CP-22). CP-4 had an abnormal karyotype containing multiple small acentric fragments or double minutes (dmin). In CP-4, FISH analysis was positive for *MYC* gene amplification, and microarray analysis further characterized the 8q24 amplified region (Figure 3a–3c). Cases CP-10 (Figure 4a) and CP-16 had aneusomies as the sole abnormality, all of which were detected by both G-banding/FISH and microarray analysis. CP-22 had a normal karyotype by G-banding analysis; however, two abnormal cell lines with heterozygous (nuc ish (D13S319x1, LAMPx2)) and homozygous (nuc ish (D13S319x0, LAMP x2)) 13q14 deletions were detected by FISH analysis (Supplementary Table S2). Microarray analysis revealed two distinct regions of copy number loss suggestive of heterozygous (13q13.3-q21.2) and homozygous (13q14.2-q14.3) deletions (Supplementary Table S2).

CP-12 had a questionable del(5)(q34q35) in all 20 G-banded cells. FISH analysis was negative for deletions at the D5S23 (5p15.2), EGR1 (5q31), and CSF1R (5q33-34) loci, and microarray was normal.

### Partially concordant results between G-banding/FISH and microarray analyses

**Figure 5:**
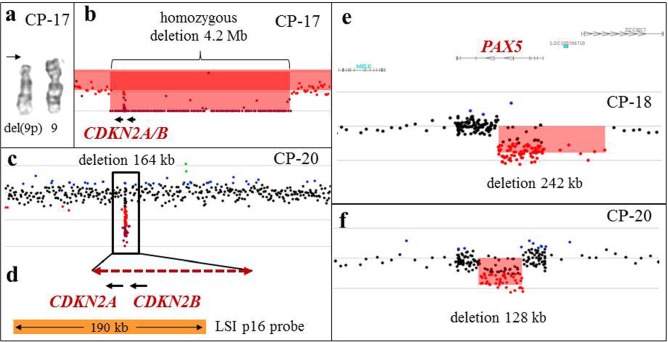
Array-CGH analysis detects cryptic aberrations of diagnostic significance **a.** A partial karyotype from CP-17, showing a deletion of the short arm of one chromosome 9 (arrow), and an apparently normal chromosome 9 homolog. **b.** Array-CGH analysis revealed a 4.2-Mb homozygous deletion spanning *CDKN2A* and *CDKN2B*, indicating that this deletion is present on what appears to be a normal chromosome 9. **c.** Array-CGH analysis of CP-20 revealed a 164-kb homozygous deletion spanning *CDKN2A* and *CDKN2B*. Aberrations of chromosomes 9 were not detected by G-banded karyotype. **d.** A magnified view of the deleted region (dashed double arrow) spanning the *CDKN2A* and *CDKN2B* genes from CP-20. The deleted segment spans a part of the LSI p16 FISH probe (Vysis; orange bar), precluding detection of this deletion by FISH analysis. **e, f.** Intragenic deletions of the *PAX5* gene in two patients with B-ALL (CP-18 and CP-20), detected by microarray analysis.

**Figure 6:**
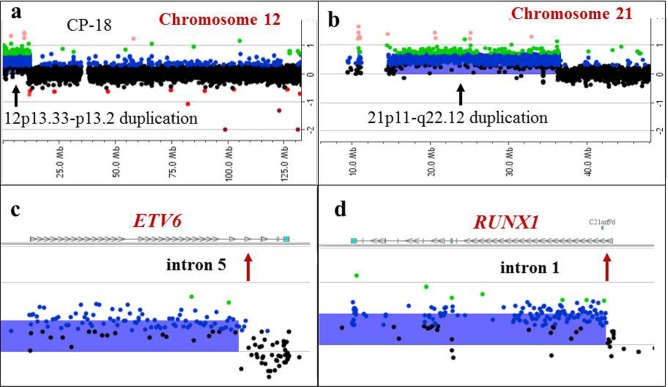
Detection of a supernumerary der(21)t(12;21) chromosome by array-CGH analysis in a patient with an apparently normal karyotype by classical cytogenetic analysis Array-CGH plots for chromosome 12 **a.** and 21 **b.** revealing gains of 12p13.33–13.2 and 21p11-q22.12. **c, d.** Magnified views showing breakpoint sites (red arrows) located in intron 5 of the *ETV6* gene (c), and intron 1 of the *RUNX1* gene (d). These results are consistent with an *ETV6/RUNX1* rearrangement and an extra der(21)t(12;21)(p13;q22) chromosome, also detected by FISH.

**Figure 7:**
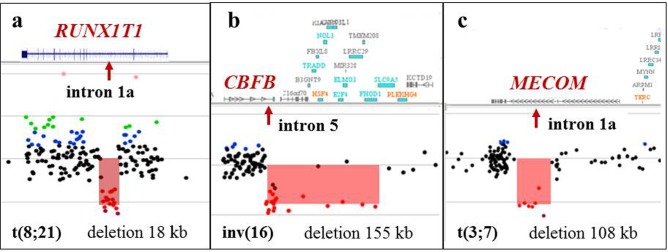
Alterations at the breakpoint sites detected by microarray analysis in apparently balanced translocations detected by classical cytogenetic analysis **a.** Array-CGH analysis in K-1 demonstrating an 18 kb deletion spanning part of intron 1a (arrow) of the *RUNX1T1* gene, a common breakpoint site associated with the *RUNX1/RUNX1T1* rearrangement. G-banded chromosome analysis revealed what appeared to be a balanced t(8;21)(q22;q22) translocation. **b.** Array-CGH analysis of CP-7 demonstrating a 155 kb deletion spanning exon 6 of the *CBFB* gene and 15 other genes downstream of *CBFB*. The proximal breakpoint of this deletion is located in intron 5 of the *CBFB* gene. G-banded analysis revealed what appeared to be a balanced inv(16)(p13q22). **c.** Microarray analysis of K-3 showed a 108 kb deletion spanning intron 1a of the *MECOM* gene in what was an apparently balanced t(3;7)(q26;q22) translocation.

In 14 cases (CP-1, CP-7, CP-8, CP-11, CP-13, CP-15, CP-17, CP-18, CP-20, CP-23, CP24, CP-25, K-1 and K-3), microarray results were consistent with previous findings by classical chromosome and/or FISH studies. Moreover, additional alterations (Supplementary Table S2) were detected in each case, including: partial monosomy for 4p and 8p (CP-24; Figure 4b); low level mosaicism for hyperdiploidy (CP-25; Figure 4c); homozygous *CDKN2A* and *CDKN2B* deletions (CP-17 and CP-20, Figure 5a–5d); heterozygous deletions of *PAX5* (CP-18 and CP-20, Figure 5e–5f); an extra derivative chromosome der(21)t(12;21) in a patient with a normal karyotype (CP-18, Figure 6); amplification of the 8q24 region containing the *MYC* gene represented by homogeneously staining regions (hsr) (CP-23, Figure 3d–3f); and submicroscopic imbalances at the translocation or inversion breakpoints (K-1, CP-7, K-3, Figure 7). CP-1 had an extra chromosome 21 in 2 of 20 metaphase cells, but interphase FISH analysis using D21S259, D21S341, and D21S342 (21q22.13-q22.2) probes was negative for an extra chromosome 21. Microarray analysis did not detect trisomy 21, but did reveal a 419-kb heterozygous deletion of the 17q11.2 chromosome region that included the *NF1* gene (Supplementary Table S2).

### Discordant results between G-banding/FISH and microarray analyses

Five of 27 cases (CP-2, CP-5, CP-6, CP-9 diagnosed with AML, and CP-14 diagnosed with CML) with balanced translocations as the sole main abnormality according to G-banding and/or FISH analysis had normal microarray results as expected.

### Detection rate for numerical and structural chromosome abnormalities

In our study, 121 abnormalities were detected by G-banding and/or FISH analyses and compared to microarray findings (Table 1). These included 78 (14 monosomies, 30 trisomies, 3 tetrasomies, 18 deletions, 1 duplication, and 12 derivative chromosomes) unbalanced abnormalities characterized by G-banding, 24 aberrations of unknown origin, and 19 balanced rearrangements. Microarray results were consistent with the results of G-banding for (62 + 8)(concordant + partially concordant) of 78 abnormalities (89.7%). This includes 13 monosomies (5 concordant + 8 partially concordant, interpreted as monosomies by classical cytogenetic studies), 27 trisomies, 3 tetrasomies, 14 deletions, 1 duplication, and 12 derivative chromosomes (Table 1). Interestingly, whole chromosome monosomies had been detected by microarray analysis in only 5 of 14 instances (36%), while in 8 of 14 cases (57%), the DNA segments from the missing chromosome were present in parts comprising derivative, marker, or double minute chromosomes.

**Table 1:** Summary of microarray results compared to classical cytogenetics (G-banding) and FISH results for 27 hematologic malignancy specimens

Aberration		Number of Abnormalities detected by G-banding/FISH	Microarray results				
			Concordant	Partially concordant	Discordant (not detected)	Additional findings (not detected by G-banding/FISH)	
						Overall	Clinically significant^&^
Unbalanced, characterized by G- banding/FISH	Whole chromosome monosomy	14	5/14 (36%)	8/14 (57%)^^^	1	3	–
	Whole chromosome trisomy	30	27/30 (87%)	–	3	5	5
	Whole chromosome tetrasomy	3	3/3 (100%)	–	–	2	–
	Deletions	18	14/18 (78%)	–	4	36	13
	Duplications	1	1/1 (100%)	–	–	16	–
	Derivative chromosomes	12	12/12 (100%)	–	–	3	2
	**Total**	**78**	**62/78 (79.5%)**	**8/78 (10.2%)**	**8/78 (10.3%)**	**65**	**20/65 (30.7%)**
Unbalanced, undetermined	Derivative chromosomes with chromatin of unknown origin	13	–	7	–	5	1
	Marker/dmin chromosomes	10	1	–	–	9*	–
	Uncertain deletion	1			1	–	–
	**Total**	**24**	**1**	**7**	**1**	**14**	**1**
Balanced rearrangements^@^ (breakpoint sites)		19 (34)	2 (3)	–	–	6 (9)	5/6

^ losses of chromosomal material have been detected by microarray, however some chromosomal segments were present on the derivative, marker or dmin chromosomes. Detailed info is given in the Supplementary Table S2.

& losses or gains encompassing genes that were reported in the literature to be involved in the pathogenesis or associated with patient's disease prognosis.

* microarray detected gains of genomic segments, elucidating chromosome origin of marker and dmin chromosomes.

@ see Table 2 for genomic loci and sizes.

**Table 2:** Genomic alterations at the breakpoints of cytogenetically balanced rearrangements

Patient	Diagnosis	Rearrangements	Abnormal G-banded cells	Abnormal cells by FISH	Rearrangement type	Alteration(s) at breakpoint site(s)	Location(s)	Size	Gene at breakpoint
CP-2	AML	t(15;17)(q24;q21)	[16/20]	[200/237]	Recurrent	–	–	–	–
CP-3	AML	t(9;11)(p22;q23)	[19/20]	Not performed	Recurrent	–	–	–	–
CP-5	AML	t(4;12)(q12;p13)	[17/20]	[223]	Recurrent	–	–	–	–
CP-7	AML	inv(16)(p13q22)	[15/20]	[173/218]	Recurrent	Loss	16q22.1	155 kb	*CBFB*, intron 5
CP-9	AML	t(16;16)(p13;q22)	[7/20]	Not performed	Recurrent	–	–	–	–
CP-14	CML	t(9;22)(q34; q11.2)	Not performed	[203/240]	Recurrent	–	–	–	–
CP-15	CML	t(9;22)(q34; q11.2), + der(22)t(9;22)	[20]	[224]	Recurrent	Loss Gain Loss Gain	9q34.11-q34.12 9q34.12-q34.3 22q11.23 22q11.1-q11.23	1.9 Mb 7.5 Mb 1.6 Mb 7.6 Mb	*ABL1*, intron 1 *BCR*, major breakpoint
CP-18	B- ALL	t(12;21)(p13;q22), + der(21)t(12;21)	[0/20]	[210/222]	Recurrent	Gain Gain	12p13.33-p13.2 21p11.2-q22.12	11.8 Mb 27 Mb	*ETV6*, intron 5 *RUNX1*, intron 1
CP-19	B- ALL	t(4;11)(q21;q23)	[17/20]	[236/242]	Recurrent	Gain	11q23.3-q25	16.6 Mb	*MLL*, intron 8
K-1	AML	t(8;21)(q22;q22)	[20]	Not performed	Recurrent	Loss	8q21.3	18 kb	*RUNX1T1*, intron1a
K-3	AML	t(3;7)(q26;q22)	[20]	Not performed	Recurrent	Loss Loss	3q26.2 7q22.1	108 kb 82 kb	*MECOM*, intron 1a
CP-6	AML	t(11;20)(p15; q11.2)	[18/20]	NA	Non-recurrent	–	–	–	–
CP-6	AML	t(3;5)(q21;q31)	[2/20]	NA	Non-recurrent	–	–	–	–
CP-6	AML	t(12;17)(p10;q10)	[2/20]	NA	Non-recurrent	–	–	–	–
CP-15	CML	t(5;6)(q13;q23)	[20]	NA	Non-recurrent	–	–	–	–
CP-19	B- ALL	t(2;15)(q31;q22)	[4/20]	NA	Non-recurrent	–	–	–	–
CP-19	B- ALL	t(5;6)(q35;p21)	[4/20]	NA	Non-recurrent	–	–	–	–
CP-19	B- ALL	t(16;17)(p13.3;q12)	[17/20]	NA	Non-recurrent	Loss Loss	16p13.3–p11.2 17p13.3-p11.2	30.3 Mb 20.7 Mb	*SRCAP*
K-3	AML	t(2;5)(p13;q33)	[20]	NA	Non-recurrent	–	–	–	–

AML, acute myeloid leukemia; CML, chronic myelogenous leukemia; B-ALL, B-cell acute lymphoblastic leukemia;NA, not applicable.

Eight of 78 (10.3%) abnormalities (Table 1), including 1 monosomy, 3 trisomies, and 4 deletions, were not detected by microarray analysis. In CP-19, monosomy 18 and trisomy for chromosomes 1, 13, and 15 were not detected by microarray analysis whereas G-banding detected +X, +1, +8, +13, +15, −18. In this case, karyotype analysis was performed on unstimulated bone marrow cultures, in contrast to microarray studies that used DNA from an uncultured specimen. In order to evaluate the level of mosaicism in cultured cells, we completed additional FISH studies using the X centromere-specific probe on interphase cells from the patient's 24-hour culture. FISH analysis showed trisomy X in 16 of 200 cells examined (8%) from a 24-hour culture, while in a 72-hour culture, additional chromosome X was observed in 10 of 20 cells (50%). Therefore, the level of abnormal cells with trisomy 1, 13, 15 and monosomy 18 *in vivo* is likely to be below the detection range (~10%) for microarray analysis.

**Figure 8:**
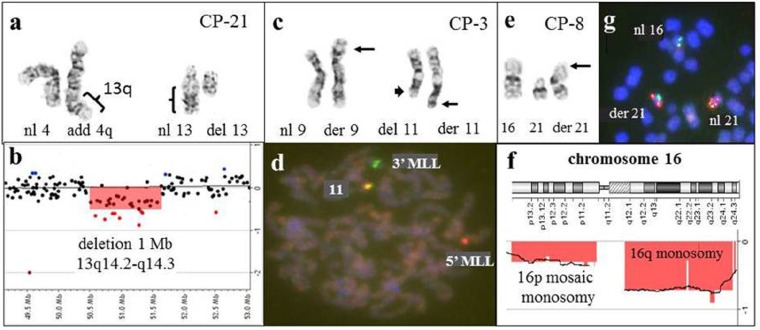
Combined results of G-banding, FISH, and microarray studies enable more accurate characterization of chromosomal aberrations in patients with hematological malignancies **a.** Partial karyotype of CP-21, showing add(4)(q35) and del(13)(q12q22), and **b.** array-CGH analysis showing a 1-Mb deletion of the 13q14.2-q14.3 chromosome region. The discrepancy between the 13q deletion size, along with the absence of genomic region gains, prompted a retrospective evaluation of the G-banded karyotype. Based on combined data, it has been concluded that the additional material on 4q is derived from 13q, and the rearrangement can be interpreted as a complex unbalanced translocation between chromosomes 4q and 13q, resulting in an interstitial 13q14 deletion. **c.** Partial karyotype of CP-3, showing the t(9;11)(p22;q23) translocation (arrows), in addition to a del(11)(q23q24) involving the other chromosome 11 (arrow head). **d.** An *MLL* break-a-part probe (11q23) was utilized to confirm the t(9;11) translocation and to confirm the 11q23-q24 deletion involving the other chromosome 11. The metaphase cell demonstrates a single red and single green signal, consistent with a split *MLL* probe and the t(9;11)(p22;q23) translocation. However, the presence of a fused yellow signal indicates that the 11q23-q24 region was inserted elsewhere in the genome and was not truly deleted. This was consistent with microarray analysis, as no deletions were detected in the 11q23-q24 region. **e.** Partial karyotype of CP-8, showing additional chromatin material on the short arm of chromosome 21. **f.** Array-CGH plot for chromosome 16, showing a loss for 16q and mosaic loss for 16p. **g.** FISH with 21q and 16p-specific probes confirmed the presence of 16p on the derivative chromosome 21.

Four deletions—del(11)(q23q24)[19] (CP-3), del(2)(q31q37)[2/20] (CP-13), del(5)(q12q33)[1] (CP-13), and del(3)(q26.2q27)[4/20] (CP-19)—were not detected by microarray analysis. These deletions might be not detected by microarray due to a low level of abnormal clones in uncultured samples, or alternatively, the chromosomal regions which appear to be deleted might be inserted into another genomic region. Thus, in CP-3 (Figure 8c–8d), the deletion identified by G-banding was not confirmed by FISH or microarray studies.

### Additional genomic alterations revealed by microarray

Microarray analysis detected 183 CNVs including losses in 94 regions, gains in 84 regions, and DNA amplification in five genomic loci (Supplementary Table S2). In addition, nine LOH segments ranging in size from 33.6 to 197.5 Mb, were detected in two of 11 cases studied by CGH + SNP design.

In 14 cases (14/27, 52%) (CP-4, CP-7, CP-8, CP-11, CP-13, CP-15, CP-17, CP-18, CP-19, CP-20, CP-23, CP-25, K-1, and K-3), microarray analysis revealed 94 additional genomic alterations. At least 65 additional genomic alterations revealed by microarray were in the regions previously characterized by classical chromosome karyotyping and/or FISH (Supplementary Table S2), 20 of which (30.7%, Table 1) had diagnostic and/or prognostic significance in patients with hematologic malignancies. For example, homozygous *CDKN2A* and *CDKN2B* deletions in two patients with B-cell acute lymphoblastic leukemia (B-ALL), which confers a poor prognosis in adult B-ALL [27], and heterozygous *PAX5* deletions in two B-ALL patients (Figure 5) were not detected by traditional chromosome studies, but were revealed by aCGH analysis. Both homozygous *CDKN2A* and *CDKN2B* deletions and heterozygous *PAX5* deletions are frequent abnormalities in pediatric and adult B-ALL [15, 28, 29], and are valuable markers for disease monitoring.

Among additional microarray findings, there were 61 cryptic alterations (61/94, 65%) that are below 10 Mb in size (Figures 2, 3, 5, 7), and 28 DNA segments (28/94, 30%) larger than 20 Mb, including 10 instances of whole chromosome aneuploidy. In CP-25 (Figure 4c), microarray analysis revealed an abnormal clone with trisomy for chromosomes 3, 5, 9, 11, 15, 19, and 21 and monosomy X, while classical cytogenetic study showed a 45,X,-X karyotype in 20 cells. Similarly, gains in the 12p13.2-p13.3 and 21p11.2-q22.12 chromosome regions, which correspond to an extra derivative chromosome, der(21)t(12;21)(p13;q22), due to *ETV6*/*RUNX1* gene rearrangement (Table 2, Figure 6), were identified, in CP-18, with normal karyotype. This highlights the advantage of evaluation of uncultured samples by microarray, as populations of malignant cells may not divide *in vitro*, under the same conditions as normal cells, and could therefore be missed by classical cytogenetic analysis.

Among our cases, microarray analysis detected genomic imbalances present as low as in 7–10% of abnormal cells.

A total of 19 apparently balanced rearrangements, including 18 translocations and one inversion, were identified by G-banding and/or FISH analysis (Table 2). Eleven rearrangements were recurrent abnormalities associated with oncogenic fusion transcripts, whereas the remaining eight were identified as non-recurrent. Balanced rearrangements are not detectable by microarray techniques; however, microarray detected genomic imbalances in six of 11 (55%) recurrent cytogenetically balanced abnormalities (Figure 7), and in one of 8 (13%) non-recurrent apparently balanced translocations.

### Combined G-banding and microarray results help to characterize complex rearrangements

In addition to the 78 abnormalities already described, 24 unbalanced rearrangements, including 13 derivative chromosomes with chromatin of undetermined origin, 10 marker chromosomes and/or double minutes, and 1 questionable deletion, were identified by G-banding analysis (Table 1). Of the 13 derivative chromosomes with chromatin of undetermined origin from five cases (CP-8, CP-21, CP-23, K-1, and K-3), five rearrangements could be further characterized on the basis of additional microarray studies. In CP-21, a microarray finding of a 1.03-Mb deletion within 13q14 and inconsistency with the reported karyotype prompted a retrospective review of the classical chromosome analysis and enabled precise characterization of the complex chromosomal rearrangement. The abnormalities add(4)(q35) and del(13)(q12q22) were reinterpreted as der(13)t(4;13)(q35;q14)del(13)(q14.2q14.3) (Figure 8a–8b). In CP-8, additional chromatin on 21p was determined to originate from 16p (Figure 8e–8f), and in CP-23, homogeneously staining regions hsr(14)(p11.2) and hsr(22)(p11.2) were determined to be composed of 8q24 (Figure 3d–3e) and 2p, respectively.

In cases with marker chromosomes and/or double minutes, microarray analysis provides the precise physical locations of amplified genomic regions, which can be further studied by FISH analysis, if necessary. For example, CP-13 had 1~4 marker chromosomes and 4~27 double minutes by G-banding (Figure 2). Microarray analysis detected multiple regions on chromosome 9 which appeared to be duplicated, triplicated, or amplified, as well as an amplified pericentromeric 20q11.2 chromosome region. The marker chromosomes and double minutes in this case are most likely to be derived from chromosome 9 and the 20q11.2 chromosome region, as no additional gains were detected by microarray analysis.

### CGH + SNP design has advantage in detection of acquired LOH regions

**Figure 9:**
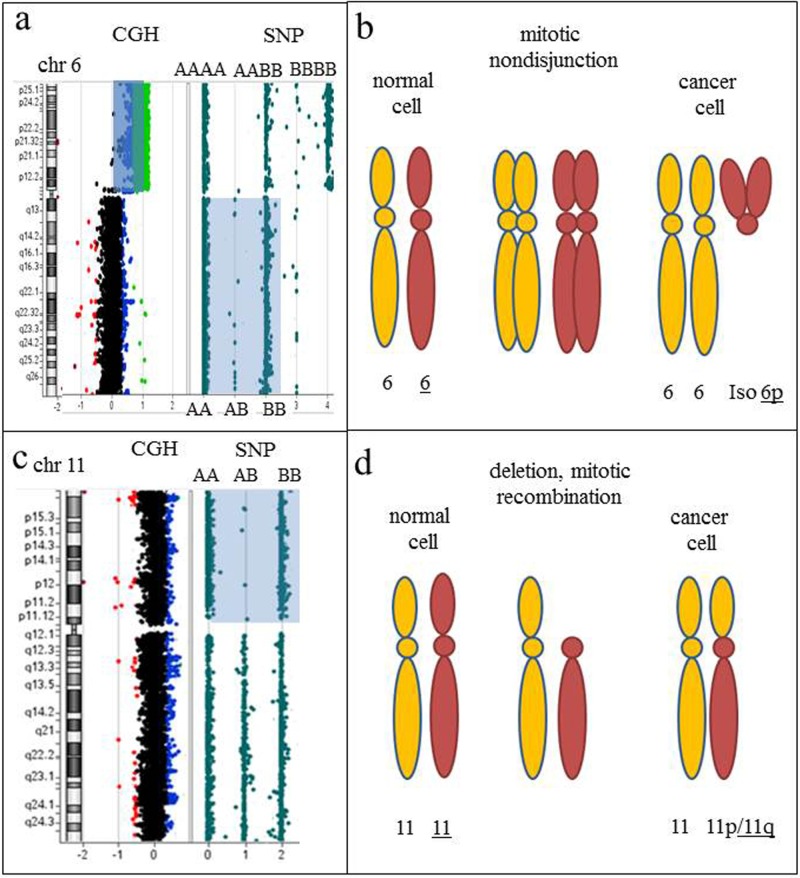
Regions of homozygosity detected by CGH+SNP array analysis point toward the nature of chromosomal rearrangements and mechanisms of clonal evolution **a.** Array-CGH plot of chromosome 6, showing a two-copy gain for all 6p-specific probes (blue shaded area). An idiogram of chromosome 6 is on the left. The SNP plot, showing a loss of heterozygosity (LOH) for the long arm of chromosome 6 (light blue shaded area), indicated by two tracks of homozygous alleles (AA and BB), is on the right. Isochromosome 6p is detected by an increased log_2_ ratio, consistent with four copies of 6p (array-CGH analysis) and three tracks of homozygous and heterozygous alleles detected by SNP analysis (AAAA, AABB, and BBBB). **b.** Proposed mechanism of clonal evolution. Mitotic nondisjunction resulting in two copies each of homologous chromosomes. Further mitotic aberrations resulting from genomic instability include uniparental isodisomy (identical copies of two chromosomes) and an isodicentric 6p chromosome (i.e., one composed of two copies of the short arm). **c.** Idiogram of chromosome 11 on the left, accompanied by CGH and SNP results on the right. Two apparently normal chromosomes 11 were detected by classical chromosome analysis and array-CGH plot, consistent with chromosome 11 disomy. LOH for the short arm of chromosome 11 is indicated by two tracks of homozygous alleles detected by SNP analysis (AA and BB). **d.** Proposed mechanism of clonal evolution. Deletion of the short arm of chromosome 11, followed by replication of the short arm of the homologous chromosome 11, resulting in identical copies of 11p (LOH).

**Figure 10:**
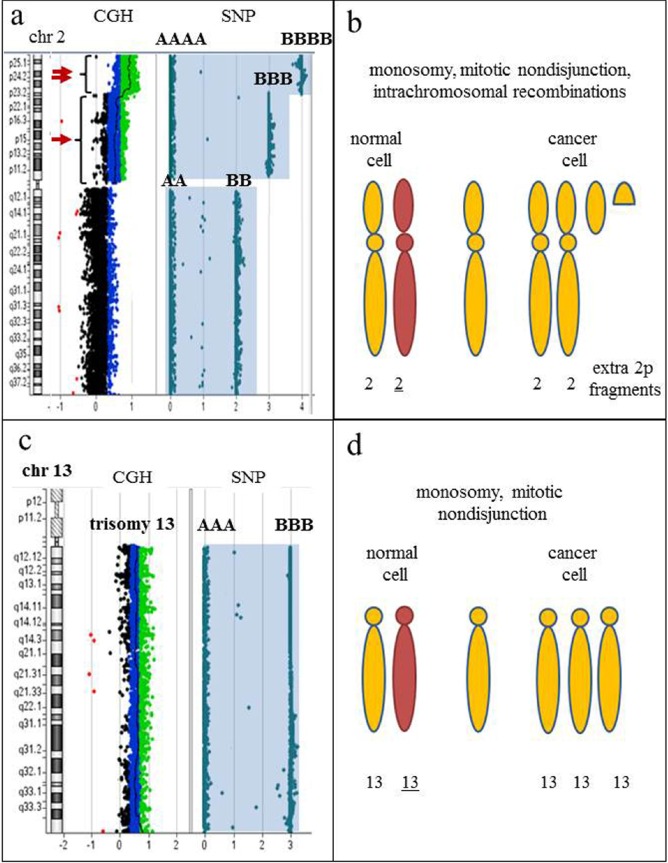
SNP-containing microarrays are essential to differentiate clones with hyperdiploidy from a doubled hypodiploid population **a.** Idiogram of chromosome 2 on the left, accompanied by CGH+SNP results on the right. Array-CGH analysis reveals a duplication (arrow) and triplication (double arrow) of the short arm of chromosome 2. Whole chromosome LOH is demonstrated by homozygous allele tracks (AA and BB; AAA and BBB; AAAA and BBBB). **b.** Proposed mechanism of clonal evolution. The initial aberration is monosomy 2, followed by mitotic nondisjunction and intrachromosomal recombination, producing extra fragments of 2p. The presence of LOH for the entire chromosome 2 indicates that numerical aberration is a primary event, while structural abnormalities are secondary changes in clonal evolution. **c.** Idiogram of chromosome 13 on the left, accompanied by CGH+SNP results on the right. Array-CGH analysis reveals trisomy 13 by an increased log_2_ ratio, and SNP array detected two homozygous allele tracks (AAA and BBB), indicating all three copies of chromosome 13 are identical. **d.** Proposed mechanism of clonal evolution. The initial aberration is monosomy 13, followed by mitotic nondisjunction, resulting in whole chromosome LOH and gain of chromosome 13. Karyotypes with gains of multiple chromosomes are commonly interpreted as hyperdiploid; however, the presence of LOH regions for multiple chromosomes strongly indicates a hypodiploid nature of the rearrangement, thus differentiating true hyperdiploidy from doubled low-diploidy or near-haploid clones and affecting prognostic significance.

A total of 11 cases (CP-1, CP-3, CP-4, CP-6, CP-11, CP-14, CP-15, CP-16, CP-17, CP-19, and CP-23) were evaluated by CGH+SNP microarray. In two cases (CP-15 and CP-23), we detected acquired copy number neutral abnormalities - loss of heterozygosity (LOH) and LOH associated with gains of chromosomal segments (Supplementary Table S2). In CP-15, trisomy X was associated with LOH, which suggests clonal evolution stemming from the loss of one X chromosome, followed by duplication of the remaining chromosome X twice. In CP-23, LOH was detected on chromosomes 2, 3, 6q, 11p, 13, 17, and 22 (Figures 9 and 10). Detection of LOH regions provided additional information toward the mechanism of clonal evolution.

## DISCUSSION

Detection of malignant clones in hematologic specimens historically has involved the study of G-banded chromosomes, which provides low-resolution evaluation of the whole-genome. Complemented with locus-specific FISH probes often used in panels, these studies remain the gold standard for detection of abnormal clones in hematologic malignancies [1]. However, the low resolution, the subjective nature of G-banding analysis, the need for dividing cells, the number of metaphase cells studied, and the limited number of FISH studies that can be performed on any given case, are considerable disadvantages when compared to high-resolution molecular-based techniques. In addition, the labor-intensive specimen processing and time-consuming demands of chromosome and FISH analysis continue to mount with an increasing volume of specimens.

High-resolution whole-genome arrays which detect copy number changes, mosaicism, and LOH (SNP-array), have revolutionized the field of clinical cytogenetics. To date, microarray analysis is recommended as the first-tier test in clinical diagnosis of patients with congenital anomalies, intellectual disability and in prenatal diagnosis of fetuses with abnormal ultrasound findings [30–31]. Microarray analysis allows chromosome evaluation at a significantly higher resolution than standard karyotype analysis, enabling detection of genomic imbalances throughout the whole genome in a single assay. The aims of our study were to evaluate the clinical utility, diagnostic yield and various technical aspects of microarray analysis in patients with hematologic malignancies.

Our group and others [17–24], have clearly demonstrated that approximately 85–90% of unbalanced clonal aberrations found by classical karyotypes are detectable by microarray analysis. G-banding analysis is beneficial in the detection of balanced chromosome alterations (translocations and inversions), examination of aberrant chromosomes and complex chromosomal rearrangements, and identification of abnormalities in related or unrelated malignant clones.

In 52% of our cases, array-CGH reveals additional genomic imbalances not seen by karyotype or FISH [17–24]. These imbalances comprised common alterations associated with specific hematologic malignancies, including deletions in the regions of *CDKN2A, CDKN2B, PAX5, NF1, ETV1, ETV6, EZH2;* amplifications of *MYC* and *KIT*; and alterations in other regions of the genome known to have diagnostic, prognostic, or treatment-related significance [27–29, 32–36]. Many clinically significant alterations are cryptic and variable in size, and may remain undiagnosed even by FISH studies with locus-specific probes. In our study, 65% of genomic regions uncovered by array-CGH were alterations less than 10 Mb in size. As shown in case CP-20 (Figure 5), a 164-kb homozygous deletion involving *CDKN2A* and *CDKN2B* spans only a part of the 190-kb LSI p16 probe, therefore, the remaining part of the probe will produce hybridization signals on both homologous chromosomes, yielding a false negative result.

Microarray analysis has greater analytical sensitivity to detect cryptic rearrangements that are below the resolution of karyotype or FISH analyses; however, it also adds value to reveal clonal abnormalities in a population of non-dividing cells. In our study, ~30% of novel alterations detected by microarray analysis were larger than 20 Mb, but were missed by G-banding due to poor chromosome morphology or a lack of abnormal clones among the population of dividing cells. In patients with hematologic malignancies, specimens comprise a mixture of neoplastic and normal cells. Regardless of the technique used, the goal of classical and molecular cytogenetic testing in hematologic cancers is to find a clone of genetically abnormal cells among the total population of cells studied. An abnormal clone is defined by classical chromosome analysis as a population composed of at least two metaphase cells of 20 studied with the same structural aberration, thus G-banding analysis has the ability to identify 10% mosaicism. FISH studies may detect as low as 3–10% of abnormal cells, depending on the number of interphase nuclei examined and the probe used. Among our samples, alterations of at least 1 Mb in size detected by FISH in 8–15% of cells were also revealed by microarray studies. Evaluation of clonal abnormalities in populations of non-dividing cells can also be achieved by FISH analysis on interphase cells; however, prior knowledge of regions of possible interest is required for targeted FISH assays. In addition, FISH assesses abnormalities at a single genomic locus and, in some cases, may yield inconclusive or borderline results. In contrast to FISH, microarray analysis utilizes data from multiple oligonucleotide probes and does not require pre-existing knowledge of possible regions of interest, adding power to reveal low-level mosaicism and cryptic alterations through the entire genome. In addition, direct testing of DNA extracted from uncultured specimens or after enrichment of the neoplastic cells rapidly provides objective data when compared to traditional G-banding analysis. Bypassing the need for dividing cells gives a more accurate assessment of *in vivo* aberrations and tumor burden, and eliminates the potential for *in vitro* culture selection. Moreover, it provides precise breakpoint locations and gene content of the affected regions.

Collective data indicate that ~40–70% [17–24] of cancer samples have acquired genomic imbalances. Our comprehensive analysis of the affected regions showed that ~30% of these alterations contain genes directly implicated in oncogenesis along with prognostic significance [27–29, 32–36], and ~45–50% of CNVs were found in patients with complex karyotypes which is consistent with global genomic instability in cancer cells. Interestingly, tumor suppressor genes known to be involved in DNA repair are commonly observed among the deleted chromosomal segments.

Microarrays enhance our ability to characterize derivative chromosomes with additional chromatin of unknown origin, genomic losses in hypodiploid clones, complex structural rearrangements, and marker chromosomes/double minutes when performed in parallel with classical cytogenetic analysis. In patient CP-23, we observed a highly abnormal karyotype which included homogenously staining regions (hsr) on chromosomes 14 and 22 (Figure 3d–3f). Homogenously staining regions and double minutes are cytogenetic hallmarks of oncogene amplification in which amplified chromosomal regions can reach a few kilobases in size that contain a single gene, or encompass a hybrid DNA sequence composed of one or more genomic regions containing multiple genes [37]. Microarray analysis revealed *MYC* amplification that localized to the hsr on chromosome 14 by subsequent FISH analysis. In such cases, microarray analysis is the most efficient approach to identify amplified genes. Similarly, in CP-4 with AML, FISH analysis identified the composition of double minutes as amplification of the 8q24 chromosome region containing *MYC*, while the region of amplification was further characterized by microarray as a 4.45 Mb segment encompassing the *TRIB1, MYC* and *PVT1* genes (Figure 3a–3c). Importantly, previous studies showed that *TRIB1*, not *MYC*, is the most likely amplified target gene in a subtype of AML/MDS [36]. This highlights the benefit of incorporating microarray analysis into the clinical setting, which would provide high-resolution analysis and precise gene content identification for proper diagnosis and a potential targeted therapy of hematologic malignancies.

Although microarrays have advantages, they will never completely replace the traditional cytogenetic testing methodologies. Microarrays cannot provide chromosomal structural composition of complex aberrations without concurrent G-banding and/or FISH studies. In our study, about 10% of cases require analysis by all the three methodologies to enhance interpretation (Figure 8). Importantly, microarray analysis cannot detect truly balanced rearrangements that are often the primary oncogenic aberration in hematologic malignancies [38–39]. However, in many instances, apparently balanced rearrangements identified by G-banding/FISH are unbalanced at the molecular level. Among our samples, 55% of recurrent balanced rearrangements had cryptic copy number alterations at the breakpoints. The custom microarray design used in our study has ample coverage within the known recurrent translocation breakpoints, which may enhance detection of imbalances at the fusion gene or its reciprocal loci. Although the clinical significance has yet to be determined, the detection of copy number changes at breakpoint sites may herald the presence of a specific rearrangement and facilitate targeted FISH testing.

Despite the large success of genomic arrays in the constitutional setting, the transition into the oncology world has proceeded slowly. As stated by Hagenkord and Chang in 2009 [40], “array-based karyotyping is now on the verge of bursting into clinical oncology, ” yet six years later, G-banding and FISH remain the sole testing methodologies for hematologic malignancies in most cytogenetics laboratories. Microarray processing is significantly automated compared to classical karyotyping, which would reduce manual labor costs in the cytogenetics laboratory. Substantial amounts of clinically relevant data have been presented in recent years that support the integration of microarray analysis into the diagnosis of hematologic malignancies [17–24, 28]. Furthermore, technical guidelines and standards for diagnosis of neoplastic disorders by microarrays were developed by the American College of Medical Genetics and Genomics [41]. Based on collective evidence, results of microarray analysis are valuable in the diagnosis and cytogenetic risk assessment in oncologic specimens. Therefore, incorporation of microarray testing into the routine clinical diagnosis of hematologic malignancies at the time of diagnosis and relapse, in conjunction with classical karyotyping, could be very beneficial. Microarray testing can be particularly useful as a replacement for FISH assays for evaluation of bone marrow samples for multiple genomic gains and losses, such as MDS and CLL FISH panels, as long as there are sufficient numbers of neoplastic cells or they are enriched in some way; for detection of cryptic alterations that are beyond the resolution of FISH analysis; for risk assessment by genomic profiling, such as deletions involving the *IKZF1, ETV6, PAX5, RB1, EBF1* genes in ALL patients [28, 29]; in samples with normal karyotype or culture failure; in cases with suspected gene amplification of unknown origin. With most hematologic diseases, timely diagnosis and management are critical; therefore, microarray testing would be beneficial and cost-effective in the clinical oncology setting, as this technology has the potential to impact clinical management, limit additional testing at different stages of disease, and improve treatment outcome.

## MATERIALS AND METHODS

### Clinical samples

This study was approved by the Institutional Review Board (IRB) at the University of Pittsburgh (PR009100456). A total of 25 specimens (17 bone marrow aspirates, 7 peripheral bloods, 1 lymph node) and two myeloblast cell lines Kasumi-1 (K-1) and Kasumi-3 (K-3) [25–26] (ATCC repository (www.atcc.org) CRL-2724, CRL-2725), with clonal chromosomal abnormalities detected on G-banding and/or FISH analysis, or normal karyotypes were selected for microarray analysis (Supplementary Tables S1 and S2). The specimens included 11 acute myeloid leukemias (AML); 4 myelodysplastic syndromes (MDS); 4 B-cell acute lymphocytic leukemias (B-ALL); 3 chronic lymphocytic leukemias (CLL); 2 chronic myeloid leukemias (CML); 2 plasma cell myelomas (PCM); and 1 polycythemia vera (PV). Clonal chromosomal abnormalities detected by G-banding and/or FISH analysis included whole chromosome aneusomies, unbalanced rearrangements, deletions, duplications, chromatin material of undetermined origin, marker chromosomes, double minutes, and apparently balanced rearrangements.

### G-banding and FISH analyses

Conventional G-banded chromosome analysis and/or FISH analyses were performed on 25 clinical samples at the Pittsburgh Cytogenetic Laboratory. A minimum of 20 metaphase cells [except CP-18 (15 cells), CP-19 (17 cells), CP-22 (18 cells), and CP-23 (15 cells)] were analyzed from 24 and/or 48-hour harvests of unstimulated bone marrow aspirate, peripheral blood, and lymph node cell cultures. In addition, four of the specimens (CP-19, CP-23, CP-24, and CP25) were also analyzed from 72-hour phorbol-12-myristate-13-acetate (PMA) stimulated cell cultures. Two peripheral blood specimens (CP-21 and CP-22) were only analyzed from 72-hour PMA-stimulated cells cultures. FISH studies were primarily performed on interphase cells, derived from 24-hour harvests of unstimulated cell cultures using Oncology-Hematology Vysis DNA probes (Abbott Laboratories, Des Plaines, IL) according to manufacturer protocol. FISH analysis and image capture was performed using Isis FISH Imaging System v5.3 software (MetaSystems, North Royalton, OH).

### Custom microarray design

To assist in the clinical diagnosis of hematological malignancies, we developed a custom genome-wide oligonucleotide microarray using Agilent 4x180K CGH and CGH+SNP designs (Agilent Inc. Santa Clara, CA). The target gene set composed of 898 genes known to be involved in carcinogenesis was derived from The Cancer Genome Atlas (TCGA; https://tcga-data.nci.nih.gov/tcga/tcgaDownload.jsp) and The Cancer Gene Census (COSMIC; http://cancer.sanger.ac.uk/cancergenome/projects/census/) databases, and a comprehensive literature review. To achieve enhanced coverage for the 898 targeted genes, we selected ~42,000 gene-specific probes with an average of one probe per 0.5–1 kb or at least 10 probes per gene. In addition, we included ~2,000 probes mapped within 28 breakpoint “hot spot” intervals associated with recurrent balanced rearrangements, with an average spacing of one probe per 300–500 bp. The remaining probes (~136,000) for the CGH-design were evenly distributed across the rest of the genome with an average spacing of one probe per 25 kb. The CGH+SNP-design comprised the same set (44,000) of gene-specific probes, ~60,000 SNP probes, and the remaining ~76,000 oligonucleotides were the backbone probes covering the genome with an average of one probe per 40 kb. Both microarray designs had the average genomic resolution 135–200 kb and an enhanced 5–10 kb resolution for targeted oncogenes and tumor suppressor genes. Detection of homozygous AA and BB and heterozygous AB alleles were performed by SNP genotyping of ~60,000 *Alu/Rsa* site-containing oligonucleotide probes included on the Agilent CGH+SNP microarray. Long stretches of homozygosity (>20 Mb) which are unlikely to represent ancestral genomic regions, and are therefore not expected to exist in the germlines of healthy individuals, were classified as acquired LOH segments. Microarray experiments and analyses were performed according to the manufacturer's protocol. Detailed information is provided in the Supplementary Data.

## SUPPLEMENTARY MATERIALS AND METHODS TABLES




