# Supplementary material for: Integration of microarray analysis into the clinical diagnosis of hematological malignancies: How much can we improve cytogenetic testing?
Source: Oncotarget. 2015 Jul 31;6(22):18845–62. doi: 10.18632/oncotarget.4586 (PMC4662459; doi:10.18632/oncotarget.4586)
Supplement: Supplementary file 1 [file oncotarget-06-18845-s001.pdf]

SUPPLEMENTARY MATERIALS AND METHODS

Microarray analysis

DNA was extracted from uncultured samples using the Puregene DNA isolation blood kit (Qiagen, Germantown, MD) according to the manufacturer's protocol. Gender-matched male or female DNA (Coriell NA12891 and NA12878) was used as reference DNA. Specimens were tested on either custom-designed aCGH or aCGH+SNP microarrays and scanned with a DNA Microarray Scanner (Agilent, Santa Clara, CA). Aberrant copy number segments and regions with loss of heterozygosity (LOH) were detected and displayed using CytoGenomics 2.0 software (Agilent). Copy number variants (CNVs), including gains and losses, were identified using the Aberration Detection Method 2 (ADM-2) algorithm, with modifications that support the determination of the clonal fraction of genomic imbalances in cancer cells. A minimum of five consecutive probes per region, with absolute average log_2 ratio of 0.3 for any given region or a minimum region of 1 Mb in size, with a minimum absolute average log_2 ratio of 0.08. CNV intervals reported in the Database of Genomic Variants (DGV; http://projects.tcag.ca/variation/) as constitutional polymorphic regions were classified as "likely benign CNVs" and excluded from further analysis.

SUPPLEMENTARY TABLES

Supplementary Table S1: Clinical and cytogenetic data for each patient included in our study

Patient	Clinical data
CP-1	The patient is a 74-year-old male with newly diagnosed acute myeloid leukemia (AML). Classical cytogenetic analysis performed on a bone marrow aspirate revealed an abnormal clone (2 cells) with trisomy 21, negative for trisomy 21 and the *MLL* gene rearrangement by FISH.
CP-2	The patient is a 2-year-old male with newly diagnosed AML. Classical cytogenetic analysis performed on a bone marrow aspirate revealed an abnormal clone with the *PML*/*RARA* gene rearrangement, confirmed by FISH. In addition, the absence of a signal for *PML* on the derivative 17 chromosome was suggestive of a cryptic deletion involving the *PML* locus.
CP-3	The patient is a 51-year-old female with newly diagnosed AML. Classical cytogenetic analysis performed on a bone marrow aspirate revealed an abnormal clone with multiple structural and numerical abnormalities (see) including the presence of the t(9;11)(p22;q23) in all cells examined.
CP-4	The patient is an 84-year-old male with newly diagnosed AML. Classical cytogenetic analysis performed on a bone marrow aspirate revealed an abnormal clone with a loss of the Y chromosome in addition to more than 30 copies of double minutes in each cell. FISH was positive for *C-MYC* gene amplification in 84.5% (180/213) of interphase cells examined, negative for *AML1* (*RUNX1*) gene amplification, and negative for the *PML*/*RARA* gene rearrangement.
CP-5	The patient is a 66-year-old male with newly diagnosed AML. Classical cytogenetic analysis performed on a peripheral blood specimen revealed an abnormal clone with the t(4;12)(q12;p13) involving *PDGFRA* (4q12) and *ETV6* (12p13).
CP-6	The patient is a 44-year-old female with newly diagnosed AML and a previous history of CNS lymphoma (CD20-positive B-cell lymphoma with plasmacytic differentiation); status, post-therapy. Classical cytogenetic analysis performed on a peripheral blood specimen revealed two related abnormal clones. The first clone had the t(11;20)(p15;q11.2). The subclone contained additional other structural abnormalities (see). FISH was negative for the *PML*/*RARA* gene rearrangement. Classical cytogenetic analysis on a concurrent bone marrow aspirate only revealed the first clone with the t(11;20) in all cells analyzed.
CP-7	This patient is a 22-month-old male with newly diagnosed AML. Classical cytogenetic analysis performed on a peripheral blood specimen revealed two related abnormal clones. The first clone had a pericentric inversion of chromosome 16. The subclone had a deletion of the long arm of chromosome 7 (see) in addition to the inv(16). Classical cytogenetic analysis on a concurrent bone marrow aspirate revealed both related abnormal clones, and FISH was positive for the *CBFB* gene rearrangement, corresponding to the inv(16) detected on chromosome analysis.
CP-8	This is a 69-year-old female patient with newly diagnosed AML. FISH was performed on a peripheral blood specimen and was negative for the *PML*/*RARA* and *BCR*/*ABL1* gene rearrangements. However, 93% of cells had loss of one signal for the *RARA* (17q21) locus. Classical cytogenetic analysis performed on a concurrent bone marrow aspirate revealed two highly complex and related clones with multiple structural and numerical abnormalities (see).
CP-9	The patient is a 71-year-old male evaluated for leukocytosis. Classical cytogenetic analysis was performed on a bone marrow aspirate to rule out AML. An abnormal clone with a translocation t(16;16)(p13;q22) was detected.
CP-10	This patient is a 49-year-old female with newly diagnosed myelodysplastic syndrome (MDS). Classical cytogenetic analysis performed on a bone marrow aspirate revealed an abnormal clone with trisomy 8 as the sole abnormality. FISH was positive for trisomy 8 in 6.6% (17/256) of interphase cells examined and negative for monosomy 7 and the 5q31, 7q31, and 20q12 deletions.
CP-11	This is an 83-year-old male with newly diagnosed MDS. Classical cytogenetic analysis performed on a bone marrow aspirate revealed an abnormal clone with an interstitial deletion of the long arm of chromosome 20, confirmed by FISH in 37% of interphase cells examined. FISH was negative for trisomy 8 and for the 5q31 and 7q31 deletions. Prior abnormal cytogenetic studies include the 20q deletion detected by FISH analysis on a peripheral blood specimen approximately 6 weeks prior to the bone marrow study described above.

(*Continued*)

Patient	Clinical data
CP-12	The patient is a 38-year-old male with a history of MDS; status, post-chemotherapy. Classical cytogenetic analysis performed on a bone marrow aspirate revealed an unusual banding pattern of the long arm of one chromosome 5, with what appears to be a small deletion involving the distal long arm. FISH was negative for deletions of 5q31 (*EGR1*), 5q33–34 (*CSF1R*), 7q31, and 20q12, monosomy 7 and trisomy 8. A previous bone marrow study, performed approximately four months earlier, showed the same chromosome 5 with questionable 5q34q35 deletion on classical cytogenetic analysis.
CP-13	This is a 79-year-old female with a history of MDS; status, post-chemotherapy. Classical cytogenetic analysis performed on a bone marrow aspirate revealed an abnormal clone with multiple structural and numerical abnormalities, including one to four marker chromosomes and 4~27 double minute chromosomes (Table I). A previous bone marrow study, performed approximately 10 months earlier, revealed similar abnormalities (Table I), with the exception of an interstitial deletion in the long arm of chromosome 2 that was detected in the most recent study.
CP-14	A 45-year-old female with a history of chronic myelogenous leukemia (CML) had positive FISH analysis for the *BCR/ABL1* gene rearrangement in 84.6% of cells analyzed. A previous FISH analysis, performed approximately 7 months earlier, was also positive for the *BCR/ABL1* gene rearrangement in 73% of interphase cells examined.
CP-15	This is a 63-year-old female with a history of CML. Classical cytogenetic analysis performed on a bone marrow aspirate revealed an abnormal clone with multiple structural and numerical abnormalities (Table I), including the t(9;22)(q34;q11.2) and an extra der(22)t(9;22) (Philadelphia chromosome). FISH analysis was positive for the *BCR/ABL1* gene rearrangement and negative for *PML/RARA* and *CBFB* gene rearrangements. However, an extra *RARA* (17q21) signal was detected which corresponds to the isochromosome 17 seen in the classical cytogenetic study. The diagnostic bone marrow aspirate study (performed approximately 8 years earlier) only revealed the t(9;22) abnormality. Since then, two classical cytogenetic studies have detected the abnormal clone described above. In addition, several FISH analyses have consistently been positive for the *BCR/ABL1* gene rearrangement accompanied by the *ASS1* gene (9q34) deletion. Quantitative real-time PCR analysis for the *BCR/ABL1* gene transcript was positive at the major CML breakpoint region.
CP-16	This is a 68-year-old female with a newly diagnosed myeloproliferative neoplasm most consistent with polycythemia vera (PV). Classical cytogenetic analysis performed on a bone marrow aspirate revealed an abnormal clone with trisomy 9.
CP-17	The patient is a 58-year-old female with newly diagnosed B-lymphoblastic leukemia (B-ALL). Classical cytogenetic analysis performed on a bone marrow aspirate revealed an abnormal clone with deletions of the short arms of one chromosome 9 and 17 in all cells analyzed.
CP-18	This is a 3-year-old male with newly diagnosed B-ALL. Classical cytogenetic analysis performed on a bone marrow aspirate showed normal karyotype. However, FISH analysis was positive for the *ETV6/RUNX1* gene rearrangement along with an extra copy of the fusion signal for the *ETV6/RUNX1* gene rearrangement in 94.6% (210/222) of interphase cells examined. FISH was negative for the extra copies of chromosomes 4, 10, and 17 and for the *BCR/ABL1* and *MLL* gene rearrangements.
CP-19	A 67-year-old female previously diagnosed with B-ALL, with *MLL* gene rearrangement and multiple structural and numerical abnormalities, detected by classical cytogenetic analysis performed on a bone marrow aspirate. FISH was positive for the *MLL* gene rearrangement in 97.5% of interphase cells examined. Most of the abnormalities seen in the current classical cytogenetic study are consistent with the two previous bone marrow aspirate studies (Table I).
CP-20	This is a 22-year-old female previously diagnosed with B-ALL. Classical cytogenetic analysis performed on a bone marrow aspirate revealed an abnormal clone with a derivative chromosome 19, resulting from an unbalanced translocation between the long arm of chromosome 1 and the short arm of chromosome 19. The diagnostic bone marrow aspirate, performed approximately 5 months earlier, revealed the same abnormal clone. In addition, FISH was positive for the *TCF3* gene rearrangement in 81% of interphase cells examined.

(*Continued*)

Patient	Clinical data
CP-21	The patient is a 46-year-old female previously diagnosed with chronic lymphocytic leukemia/small lymphocytic lymphoma (CLL/SLL) by flow cytometry. Classical cytogenetic analysis performed on a peripheral blood specimen revealed an abnormal clone with additional material on the long arm of chromosome 4 and an interstitial deletion of the long arm of chromosome 13. FISH was positive for the 13q14.3 deletion in 33.2% of interphase cells examined and negative for trisomy 12, *IGH* gene rearrangements, and deletions involving the *MYB*, *ATM*, and *TP53* genes.
CP-22	This is a 68-year-old female previously diagnosed with CLL/SLL. FISH performed on a peripheral blood specimen was positive for the 13q14.3 deletion in 57.1% of interphase cells examined and negative for trisomy 12, *IGH* gene rearrangements, and deletions involving the *MYB*, *ATM*, and *TP53* genes. Classical cytogenetic analysis on the same specimen showed normal karyotype.
CP-23	This is a 60-year-old male previously diagnosed with CLL/SLL. Classical cytogenetic analysis on a lymph node specimen revealed an abnormal clone with multiple structural and numerical abnormalities, including homogeneously staining regions on the short arms of chromosome 14 and 22 (Table I). FISH studies were negative for *EWSR1* and *FOX01* (*FKHR*) gene rearrangements. The abnormal clone in this study was not observed in the previous studies. Abnormal related clones described in previous studies (Table I) were not identified in the above lymph node study by classical cytogenetic analysis.
CP-24	This is a 66-year-old male with previously diagnosed plasma cell myeloma (PCM); status, post–peripheral blood transplant. Classical cytogenetic analysis performed on a bone marrow aspirate revealed a hyperdiploid clone. FISH was positive for extra signals for chromosomes 5, 7, 9, and 11 and negative for the 13q14.3 deletion, *TP53* gene deletion, and *IGH* gene rearrangement.
CP-25	This is an 88-year-old female with newly diagnosed PCM. Classical cytogenetic analysis revealed monosomy for chromosome X in 3/20 cells as the sole abnormality. FISH was positive for an extra chromosome 5 (6.7%) and chromosome 11 (7.4%) signals and negative for deletions of 13q14.3 and *TP53* (17p13.1). FISH studies were also negative for trisomies 7 and 9 and gene rearrangements involving *IGH* and *IGH*/*CCND1*.
K-1	The cell line was established from the peripheral blood of a 7-year-old Japanese boy with acute myeloid leukemia (AML) and t(8;21)(q22;q22) (Asou H. et al, 1991).
K-3	The cell line was established from the blast cells of a 57 year old male Japanese patient with myeloperoxidase-negative acute leukemia and t(3;7)(q27;q22)(Asou H. et al, 1996).

Supplementary Table S2: Classical cytogenetic (G-banding), FISH, and microarray analysis results in 27 samples with hematologic malignancies
