# Supplementary material for: Integration of microarray analysis into the clinical diagnosis of hematological malignancies: How much can we improve cytogenetic testing?
Source: Oncotarget. 2015 Jul 31;6(22):18845–62. doi: 10.18632/oncotarget.4586 (PMC4662459; doi:10.18632/oncotarget.4586)
Supplement: Supplementary file 2 [file oncotarget-06-18845-s002.docx]

**Table S2.** Classical cytogenetic (G-banding), FISH, and microarray analysis results in 27 samples with hematologic malignancies.

| **Sample** | **Gender/Age, yrs** | **Dx** | **Specimen type** | **Karyotype and FISH results** | **Microarray** | **Chr** | **Cytoband** | **Start** | **Stop** | **Size, bp** | **Copy number** | **Comments** | **Total Genes** | **Critical Genes/regions** |
| --- | --- | --- | --- | --- | --- | --- | --- | --- | --- | --- | --- | --- | --- | --- |
| CP-1 | M/74 | AML | BM | 47,XY,+21[2]/46,XY[19] nuc ish(MLLx2)[200], (D21S259,D21S341,D21S342)x2[200] | CGH+SNP | 17 | q11.2 | 29,320,520 | 29,739,521 | 419,001 | Loss | Mosaic,  additional findings | 9 | *NF1* |
| CP-2 | M/2 | AML | BM | 46,XY,t(15;17)(q24;q21)[16]/46,XY[4]  nuc ish(PMLx2,RARAx3)(PML con RARAx1)[200/237] | CGH | None |  |  |  |  |  |  |  |  |
| CP-3 | F/51 | AML | BM | 47,XX,dup(1)(q21q43),der(5)t(1;5) (q21;p15),+8,t(9;11)(p22;q23),der(10) t(1;10)(q21;q25),del(11)(q23q24) [cp19]/46,XX[1] | CGH+SNP | 1 | q21.1 - q44 | 144,009,907 | 249,197,762 | 105,187,856 | Gain | dup(1) | 1,656 |  |
|  |  |  |  |  |  | 8 | p23.3 - q24.3 | 194,625 | 146,293,435 | 146,098,811 | Gain | Trisomy 8 | 1,320 |  |
| CP-4 | M/84 | AML | BM | 45,X,-Y,>30dmin[20].nuc ish (C-MYC amp)[180/213],(RUNX1x2) [200],(PML,RARA)x2[215] | CGH | 8 | q24.13 | 125,635,650 | 126,164,858 | 529,209 | Loss | additional findings | 9 |  |
|  |  |  |  |  |  | 8 | q24.13 - q24.21 | 126,206,094 | 130,661,197 | 4,455,104 | Amp | dmin | 22 | *MYC, PVT1* |
|  |  |  |  |  |  | Y | p11.32 - q11.23 | 13,303 | 59,031480 | 59,018,178 | Loss | -Y | 53 |  |
| CP-5 | M/66 | AML | PB | 46,XY,t(4;12)(q12;p13)[17]/46,XY[3]  nuc ish(FIP1L1,CHIC2,PDGFRA)x2,  (FIP1L1-CHIC2 sep PDGFRAx1) [200/202],(ETV6x2~5)(5'ETV6 sep 3'ETV6x1)[223] | CGH | None |  |  |  |  |  |  |  |  |
| CP-6 | F/44 | AML | PB | 46,XX,t(11;20)(p15;q11.2)[18]/46, idem,t(3;5)(q21;q31),t(12;17)(p10; q10)[2].nuc ish(PML,RARA)x2[222] | CGH+SNP | None |  |  |  |  |  |  |  |  |
| CP-7 | M/<2 | AML | PB | 46,XY,inv(16)(p13q22)[15]/46,idem, del(7)(q31q36)[5]  **Previous BM**: 46,XY,inv(16)(p13q22)[14]/46, idem,del(7)(q31q36)[5]/46,XY[1].nuc ish(CBFBx2)(5’CBFB sep 3’CBFBx1) [173/218],(EGR1,D5S23,D5S721)x2 [200],(D7Z1,D7S486)x2[200],(D8Z2,D20S108)x2[203] | CGH | 7 | q31.31 - q36.2 | 119,519,533 | 152,785,120 | 33,265,587 | Loss | del(7) | 514 | 7q (EZH2) deletion |
|  |  |  |  |  |  | 16 | q22.1 | 67,121,928 | 67,276,994 | 155,066 | Loss | additional findings inv(16) breakpoint | 16 |  |
| **Sample** | **Gender/Age, yrs** | **Dx** | **Specimen type** | **Karyotype and FISH results** | **Microarray** | **Chr** | **Cytoband** | **Start** | **Stop** | **Size, bp** | **Copy number** | **Comments** | **Total Genes** | **Critical Genes/regions** |
| CP-8 | F/69 | AML | PB | nuc ish(PMLx2,RARAx1~2)[215], (ABL1,BCR)x2[226]  **Previous B**M: 43,XX,add(1)(q25),der(5)t(5;12) (q12;q23),der(12;17)(?::12q23→12q10: :17q10→17qter),-16, der(17;21)(17pter→17p10::21q10→ 21q22::?),+mar1[10]/43~44,idem,del(11) (p13p15),-12,-17,-18,add(21) (p11.2),-22,+mar1x1~2,+mar2,+mar3[cp17] | CGH | 1 | q25.3 - q31.3 | 183,882,931 | 198,142,793 | 14,259,862 | Loss | der(1)/add(1)(q25) | 80 |  |
|  |  |  |  |  |  | 1 | q32.1 | 202,346,877 | 203,876,503 | 1,529,626 | Gain | additional findings | 44 |  |
|  |  |  |  |  |  | 1 | q32.1 - q44 | 206,683,140 | 249,197,762 | 42,514,622 | Loss | der(1)/add(1)(q25) | 557 |  |
|  |  |  |  |  |  | 5 | q11.1 - q11.2 | 49,621,217 | 53,111,368 | 3,490,151 | Loss | der(5) | 20 |  |
|  |  |  |  |  |  | 5 | q14.1 - q14.3 | 79,325,029 | 85,889,412 | 6,564,383 | Loss | der(5) | 51 |  |
|  |  |  |  |  |  | 5 | q21.1 - q21.3 | 100,143,286 | 107,787,159 | 7,643,873 | Loss | der(5) | 22 |  |
|  |  |  |  |  |  | 5 | q23.3 - q35.3 | 127,601,654 | 180,712,263 | 53,110,609 | Loss | der(5) | 701 | 5q (*EGR1*) deletion, *NPM1* |
|  |  |  |  |  |  | 11 | p15.4 - p15.3 | 3,043,105 | 12,580,691 | 9,537,586 | Loss | additional findings | 278 |  |
|  |  |  |  |  |  | 11 | p13 | 34,391,877 | 34,899,898 | 508,021 | Loss | additional findings | 4 |  |
|  |  |  |  |  |  | 12 | p12.3 - p12.1 | 15,297,997 | 22,714,240 | 7,416,243 | Loss | der(12;17) | 59 |  |
|  |  |  |  |  |  | 12 | p12.1 - p11.22 | 22,739,028 | 27,814,851 | 5,075,823 | Loss | der(12;17) | 37 |  |
|  |  |  |  |  |  | 12 | p11.22 - p11.21 | 27,832,770 | 32,524,361 | 4,691,591 | Loss | der(12;17) | 47 |  |
|  |  |  |  |  |  | 12 | q15 - q22 | 71,154,496 | 94,580,414 | 23,425,918 | Loss | additional findings | 141 |  |
|  |  |  |  |  |  | 16 | p13.3 - p11.1 | 100,293 | 35,204,414 | 35,104,121 | Loss | additional findings | 722 | *PALB2 (FANCN)* |
|  |  |  |  |  |  | 16 | q11.2 - q24.3 | 46,500,741 | 90,163,114 | 43,662,373 | Loss | segmental monosomy 16 | 545 | *FANCA* |
|  |  |  |  |  |  | 17 | p13.1 - p11.2 | 9,530,075 | 17,830,301 | 8,300,226 | Loss | additional findings | 120 |  |
|  |  |  |  |  |  | 17 | q11.2 - q24.3 | 26,109,698 | 67,126,904 | 41,017,206 | Loss | monosomy 17q | 916 | *NF1, BRCA1* |
|  |  |  |  |  |  | 17 | q12-q24.2 | 36,552,858 | 64,311,564 | 27,758,706 | Loss | additional findings | 640 | *BRCA1* |
|  |  |  |  |  |  | 18 | p11.32 - p11.21 | 69,754 | 11,982,981 | 11,913,227 | Loss | segmental monosomy 18 | 104 |  |
|  |  |  |  |  |  | 18 | q11.2 | 21,263,612 | 23,262,808 | 1,999,196 | Loss | segmental monosomy 18 | 16 |  |
|  |  |  |  |  |  | 18 | q12.1 - q12.2 | 31,333,305 | 35,122,247 | 3,788,942 | Gain | segmental monosomy 18 | 27 |  |
|  |  |  |  |  |  | 18 | q12.3 | 38,232,934 | 42,316,541 | 4,083,607 | Loss | segmental monosomy 18 | 12 |  |
|  |  |  |  |  |  | 18 | q21.2 - q21.32 | 50,162,137 | 56,416,452 | 6,254,315 | Gain | segmental monosomy 18 | 40 |  |
|  |  |  |  |  |  | 21 | q11.2 - q21.3 | 15,569,395 | 31,235,085 | 15,665,690 | Loss | segmental monosomy 21 | 99 |  |
|  |  |  |  |  |  | 21 | q21.2-q21.3 | 26,784,615 | 27,801,971 | 1,017,356 | Loss | segmental monosomy 21 | 12 |  |
|  |  |  |  |  |  | 22 | q11.21 - q12.3 | 19,694,673 | 33,440,680 | 13,746,007 | Gain | Mosaic | 413 |  |
|  |  |  |  |  |  | 22 | q13.1 | 38,160,726 | 39,449,704 | 1,288,978 | Loss | Mosaic | 41 |  |
|  |  |  |  |  |  | 22 | q13.1 - q13.2 | 39,485,954 | 41,497,032 | 2,011,078 | Gain | Mosaic | 46 |  |
| **Sample** | **Gender/Age, yrs** | **Dx** | **Specimen type** | **Karyotype and FISH results** | **Microarray** | **Chr** | **Cytoband** | **Start** | **Stop** | **Size, bp** | **Copy number** | **Comments** | **Total Genes** | **Critical Genes/regions** |
| CP-9 | M/71 | AML | BM | 46,XY,t(16;16)(p13;q22)[7]/46,XY[13] | CGH | None |  |  |  |  |  |  |  |  |
| CP-10 | F/49 | MDS | BM | 47,XX,+8[2]/46,XX[18]  nuc ish (D8Z2x3,D20S108x2)[17/256], (EGR1,D5S23)x2[223],(D7Z1,D7S486)x2[234] | CGH | 8 | p23.3 - q24.3 | 73,810 | 146,231,971 | 146,158,162 | Gain | Trisomy 8 | 1,320 |  |
| CP-11 | M/83 | MDS | BM | 46,XY,del(20)(q11.2q13.3)[13]/ 46,XY[7].nuc ish(D20S108x1)[87/235]  **Previous PB:**  nuc ish(D8Z2x2,D20S108x1) [46/214],(EGR1,D5S23)x2[222], (D7Z1,D7S486)x2[204] | CGH+SNP | 6 | q23.3 | 135,256,840 | 136,959,418 | 1,702,579 | Loss | additional findings | 18 |  |
|  |  |  |  |  |  | 20 | q11.22 - q13.2 | 33,398,645 | 54,967,603 | 21,568,959 | Loss | del(20) | 315 | 20q deletion |
| CP-12 | M/38 | MDS | BM | 46,XY,?del(5)(q34q35)[20]. ish 5p15.2(D5S23+),5q31(EGR1+), 5q33q34(CSF1R+)  **Previous BM:**  46,XY[20].nuc ish(EGR1,D5S23)x2 [201],(D7Z1,D7S486)x2[206], (D8Z2,D20S108)x2[210] | CGH | None |  |  |  |  |  |  |  |  |
| **Sample** | **Gender/Age, yrs** | **Dx** | **Specimen type** | **Karyotype and FISH results** | **Microarray** | **Chr** | **Cytoband** | **Start** | **Stop** | **Size, bp** | **Copy number** | **Comments** | **Total Genes** | **Critical Genes/regions** |
| CP-13 | F/78 | MDS | BM | 46~48,XX,del(2)(q31q37),del(5) (q12q33),del(7)(q22q36),+8,-9, -20,+1~4mar,+4~27dmin[cp20]  **Previous BM:**  47,XX,del(5)(q12q33), +8[2]/46~48,XX,del(7)(q22q36),+8, -9,-20,+1~3mar,5~20dmin[19]/46,XX[2] | CGH | 7 | q21.3 - q36.3 | 95,556,472 | 159,125,676 | 63,569,204 | Loss | del(7) | 862 | 7q (*EZH2*) deletion |
|  |  |  |  |  |  | 8 | p23.3 - q24.3 | 176,717 | 146,293,435 | 146,116,718 | Gain | Trisomy 8 | 1,321 |  |
|  |  |  |  |  |  | 9 | p24.3 | 611,561 | 2,141,830 | 1,530,269 | Gain | additional findings complex | 8 |  |
|  |  |  |  |  |  | 9 | p24.3 - p24.2 | 2,171,692 | 2,805,636 | 633,944 | Gain |  | 5 |  |
|  |  |  |  |  |  | 9 | p24.2 - p24.1 | 2,805,637 | 5,673,178 | 2,867,541 | Gain |  | 37 |  |
|  |  |  |  |  |  | 9 | p24.1 - p23 | 5,853,483 | 12,569,905 | 6,716,422 | Gain |  | 27 |  |
|  |  |  |  |  |  | 9 | p23 - p22.3 | 12,589,870 | 14,986,741 | 2,396,871 | Loss |  | 16 |  |
|  |  |  |  |  |  | 9 | p22.3 | 15,040,963 | 16,527,269 | 1,486,306 | Gain |  | 13 |  |
|  |  |  |  |  |  | 9 | p22.3 - p22.2 | 16,543,419 | 18,262,004 | 1,718,585 | Loss |  | 9 |  |
|  |  |  |  |  |  | 9 | p22.1 | 18,674,394 | 19,480,337 | 805,943 | Gain |  | 13 |  |
|  |  |  |  |  |  | 9 | p22.1 - p21.3 | 19,514,928 | 20,742,255 | 1,227,327 | Gain |  | 10 |  |
|  |  |  |  |  |  | 9 | p21.3 | 20,758,066 | 21,055,004 | 296,938 | Gain |  | 2 |  |
|  |  |  |  |  |  | 9 | p21.3 | 21,089,892 | 22,537,069 | 1,447,177 | Loss |  | 39 | *CDKN2A, CDKN2B* |
|  |  |  |  |  |  | 9 | p21.3 - p21.2 | 22,555,507 | 25,688,757 | 3,133,250 | Gain |  | 8 |  |
|  |  |  |  |  |  | 9 | p21.2 - p11.2 | 25,702,861 | 44,180,151 | 18,477,290 | Loss |  | 291 | *FANCG, PAX5* |
|  |  |  |  |  |  | 9 | q21.11 | 70,984,481 | 72,042,508 | 1,058,027 | Loss |  | 12 |  |
|  |  |  |  |  |  | 9 | q21.11 - q21.12 | 72,083,648 | 73,362,380 | 1,278,732 | Gain |  | 11 |  |
|  |  |  |  |  |  | 9 | q21.12 - q21.13 | 73,389,763 | 76,130,938 | 2,741,175 | Loss |  | 23 |  |
|  |  |  |  |  |  | 9 | q21.13 | 76,130,939 | 76,588,336 | 457,397 | Gain |  | 2 |  |
|  |  |  |  |  |  | 9 | q21.13 | 76,588,337 | 78,030,991 | 1,442,654 | Loss |  | 9 |  |
|  |  |  |  |  |  | 9 | q21.13 | 78,030,992 | 79,003,616 | 972,624 | Gain |  | 5 |  |
|  |  |  |  |  |  | 9 | q21.13 - q21.31 | 79,003,617 | 81,400,579 | 2,396,962 | Loss |  | 21 |  |
|  |  |  |  |  |  | 9 | q21.31 | 81,400,580 | 81,484,152 | 83,572 | Gain |  | 0 |  |
|  |  |  |  |  |  | 9 | q21.31 - q34.3 | 81,484,153 | 141,122,114 | 59,637,961 | Loss |  | 834 | *FANCC* |
|  |  |  |  |  |  | 11 | p15.5 - q25 | 217,046 | 134,934,196 | 134,717,150 | Loss | additional findings Monosomy 11 | 2,137 |  |
|  |  |  |  |  |  | 12 | p13.31 - p12.3 | 9,687,840 | 15,102,646 | 5,414,806 | Loss | additional findings complex | 127 | *CDKN1B, ETV6* |
|  |  |  |  |  |  | 16 | p13.3 - q24.3 | 100,293 | 90,163,114 | 90,062,821 | Loss | additional findings Monosomy 16 | 1,267 |  |
|  |  |  |  |  |  | 20 | p13 - p11.1 | 80,198 | 25,846,196 | 25,765,998 | Loss | segmental monosomy 20 | 310 |  |
|  |  |  |  |  |  | 20 | q11.21 | 29,611,849 | 31,163,149 | 1,551,300 | Amp | additional findings | 47 |  |
|  |  |  |  |  |  | 20 | q11.21 - q13.2 | 31,280,132 | 52,685,594 | 21,405,462 | Loss | segmental monosomy 20 | 353 | 20q deletion |
|  |  |  |  |  |  | X | p22.33 - q28 | 63,303 | 154,929,279 | 154,865,976 | Loss | additional findings Monosomy X | 1,682 |  |
|  |  |  |  |  |  | X | q13.1 - q21.32 | 71,678,915 | 93,294,027 | 21,615,112 | Loss | additional findings | 159 |  |
| **Sample** | **Gender/Age, yrs** | **Dx** | **Specimen type** | **Karyotype and FISH results** | **Microarray** | **Chr** | **Cytoband** | **Start** | **Stop** | **Size, bp** | **Copy number** | **Comments** | **Total Genes** | **Critical Genes/regions** |
| CP-14 | F/44 | CML | PB | nuc ish(ABL1,BCR)x3(ABL1 con BCRx2)[203/240]  **Previous PB:**  nuc ish(ABL1,BCR)x3(ABL1 con BCRx2)[149/204] | CGH+SNP | None |  |  |  |  |  |  |  |  |
| CP-15 | F/55 | CML | BM | 52,XX,+X,t(5;6)(q13;q23),+8,+8, t(9;22)(q34;q11.2),+14,i(17)(q10),+19, +der(22)t(9;22)(q34;q11.2)[20]. nuc ish(ABL1,BCR)x3(ABL1 con BCRx2)[224],(PMLx2,RARAx2~3) [133/226],(CBFBx2)[241]  **Previous BM**: 46,XX,t(9;22)(q34;q11.2)[20]  **Previous BM**: 46,XX,t(9;22)(q34;q11.2)[14]/ 49,idem,+8,+14,+19[6]  **Previous PB**: 52,XX,+X,t(5;6)(q13;q23),+8,+8, t(9;22)(q34;q11.2),+14,i(17)(q10),+19, +der(22)t(9;22)(q34;q11.2)[cp20] | CGH+SNP | 7 | p21.3 | 8,751,128 | 9,111,326 | 360,199 | Gain | additional findings | 2 |  |
|  |  |  |  |  |  | 8 | p23.3 - q24.3 | 194,625 | 146,293,435 | 146,098,811 | Gain | Tetrasomy 8 | 1,320 |  |
|  |  |  |  |  |  | 9 | q34.11 - q34.12 | 131,674,303 | 133,590,333 | 1,916,031 | Loss | additional findings t(9;22) breakpoint | 32 | *ABL1* |
|  |  |  |  |  |  | 9 | q34.12 - q34.3 | 133,593,889 | 141,122,114 | 7,528,226 | Gain | der(22)t(9;22) | 207 | *ABL1, NUP214* |
|  |  |  |  |  |  | 10 | q26.13 | 124,347,870 | 124,351,781 | 3,912 | Loss | additional findings | 1 |  |
|  |  |  |  |  |  | 14 | q11.2 - q32.33 | 19,100,682 | 107,281,934 | 88,181,253 | Gain | Trisomy 14 | 1,497 |  |
|  |  |  |  |  |  | 17 | p13.3 - p11.2 | 48,858 | 18,913,229 | 18,864,372 | Loss | idic(17)(p11.2) | 452 | *TP53* |
|  |  |  |  |  |  | 17 | p11.2 - q25.3 | 19,143,173 | 81,085,674 | 61,942,502 | Gain | idic(17)(p11.2) | 1,291 |  |
|  |  |  |  |  |  | 19 | p13.3 - q13.43 | 277,373 | 59,057,101 | 58,779,729 | Gain | Trisomy 19 | 2,026 |  |
|  |  |  |  |  |  | 22 | q11.1 - q11.23 | 16,054,691 | 23,619,676 | 7,564,986 | Gain | der(22)t(9;22) | 323 | *BCR* |
|  |  |  |  |  |  | 22 | q11.23 | 23,634,870 | 25,186,768 | 1,551,899 | Loss | additional findings t(9;22) breakpoint | 57 | *BCR* |
|  |  |  |  |  |  | X | p22.33 - q28 | 63,303 | 154,908,471 | 154,845,169 | Gain | Trisomy X | 1,682 |  |
|  |  |  |  |  |  | X | p22.33 - q28 | 492,888 | 155,158,531 | 154,665,644 | LOH | additional findings | 1,680 |  |
| CP-16 | F/68 | MPN (PV) | BM | 47,XX,+9[3]/46,XX[17] | CGH+SNP | 9 | p24.3 - q34.3 | 163,131 | 141,122,114 | 140,958,984 | Gain | Trisomy 9 | 1,504 |  |
| CP-17 | F/58 | B-ALL | BM | 46,XX,del(9)(p11.2),del(17)(p12)[20] | CGH+SNP | 9 | p24.3 - p11.2 | 163,131 | 47,212,321 | 47,049,191 | Loss | del(9) | 516 | *JAK2, CDKN2A, CDKN2B, FANCG, PAX5* |
|  |  |  |  |  |  | 9 | p21.3 - p21.2 | 21,658,291 | 25,779,281 | 4,120,991 | Loss hmz | additional findings | 18 | *CDKN2A, CDKN2B* |
|  |  |  |  |  |  | 10 | q23.1 | 84,325,725 | 84,974,010 | 648,286 | Loss | additional findings | 1 |  |
|  |  |  |  |  |  | 17 | p13.3 - p11.2 | 48,858 | 18,913,229 | 18,864,372 | Loss | del(17) | 452 | *TP53* |
| **Sample** | **Gender/Age, yrs** | **Dx** | **Specimen type** | **Karyotype and FISH results** | **Microarray** | **Chr** | **Cytoband** | **Start** | **Stop** | **Size, bp** | **Copy number** | **Comments** | **Total Genes** | **Critical Genes/regions** |
| CP-18 | M/3 | B-ALL | BM | 46,XY[15].nuc ish(ETV6x3,RUNX1 x4) (ETV6 con RUNX1x2)[210/222], (D4Z1,D10Z1,D17Z1)x2[200], (ABL1,BCR)x2[239],(MLLx2)[214] | CGH | 9 | p13.2 | 36,930,122 | 37,172,365 | 242,243 | Loss | additional findings | 4 | *PAX5* |
|  |  |  |  |  |  | 12 | p13.33 - p13.2 | 162,848 | 12,000,975 | 11,838,127 | Gain | additional findings der(21)t(12;21) | 282 | *ETV6* |
|  |  |  |  |  |  | 21 | p11.2 - q22.12 | 9,412,632 | 36,414,104 | 27,001,472 | Gain | additional findings der(21)t(12;21) | 258 | *RUNX1* |
| CP-19 | F/67 | B-ALL | BM | 45~50,XX,+X,+1,t(2;15)(q31;q22), del(2)(p21p23),del(3) (q26.2q27),t(4;11)(q21;q23),t(5;6) (q35;p21),+8,+13,+15,t(16;17)  (p13.3;q12),-18[cp17]  nuc ish(5'MLLx2, 3'MLLx2~3)(5'MLL con 3'MLLx1)[236/242]  **Previous BM:** 46,XX,t(4;11)(q21;q23),del(17) (p11.2)[9]/47,sl,+8[6]/48~49,sdl1,+X,+X[cp7]  nuc ish(5’MLLx2,3’MLLx2~3)(5’MLL con 3’MLLx1)[202/215],(4p11-q11,10p11.1-q11.1, D17Z1)x2 [200],(ABL1,BCR)x2[204], (ETV6,RUNX1)x2[218]  **Previous BM:**  45,XX,t(2;15)(q31;q22),t(4;11) (q21;q23),t(5;6)(q35;p21),der(5) t(5;17)(q31;q21),add(16)(p13.3),-17[cp20] | CGH+SNP | 2 | p24.2 - p21 | 18,075,296 | 42,405,823 | 24,330,528 | Loss | del(2) | 253 |  |
|  |  |  |  |  |  | 8 | p23.3 - q24.3 | 73,810 | 146,293,435 | 146,219,626 | Gain | Mosaic trisomy 8 | 1,322 |  |
|  |  |  |  |  |  | 9 | p21.3 - p21.1 | 23,348,535 | 32,759,755 | 9,411,221 | Gain | additional findings | 50 |  |
|  |  |  |  |  |  | 11 | q23.3 - q25 | 118,354,000 | 134,934,196 | 16,580,197 | Gain | additional findings t(4;11) breakpoint | 238 |  |
|  |  |  |  |  |  | 16 | p13.3 - p11.2 | 377,905 | 30,724,008 | 30,346,104 | Loss | additional findings t(16;17) breakpoint | 567 | *PALB2* (*FANCN*) |
|  |  |  |  |  |  | 17 | p13.3 - p11.2 | 48,858 | 20,812,846 | 20,763,989 | Loss | additional findings t(16;17) breakpoint | 516 | *TP53* |
|  |  |  |  |  |  | X | p22.33 - q28 | 63,303 | 154,908,471 | 154,845,169 | Gain | Mosaic  trisomy X | 1,682 |  |
| CP-20 | F/22 | B-ALL | BM | 46,XX,der(19)t(1;19)(q23;p13.3)[4]/ 46,XX[16] **Previous BM**: 46,XX,der(19)t(1;19)(q23;p13.3) [3]/46,XX[16].nuc ish(5’TCF3x2,3’TCF3x1)(5’TCF3 con 3’TCF3x1)[185/227] | CGH | 1 | q23.3 - q44 | 164,651,503 | 249,168,732 | 84,517,229 | Gain | Mosaic der(19)t(1;19) | 1,055 | *PBX1*, intron 2 |
|  |  |  |  |  |  | 9 | p21.3 | 21,905,380 | 22,070,042 | 164,662 | Loss hmz | additional findings | 7 | *CDKN2A, CDKN2B* |
|  |  |  |  |  |  | 9 | p13.2 | 36,862,567 | 36,990,522 | 127,955 | Loss | additional findings | 3 | *PAX5* |
|  |  |  |  |  |  | 19 | p13.3 | 266,117 | 1,609,660 | 1,343,543 | Loss | Mosaic der(19)t(1;19) | 71 | *TCF3* |
| **Sample** | **Gender/Age, yrs** | **Dx** | **Specimen type** | **Karyotype and FISH results** | **Microarray** | **Chr** | **Cytoband** | **Start** | **Stop** | **Size, bp** | **Copy number** | **Comments** | **Total Genes** | **Critical Genes/regions** |
| CP-21 | F/44 | CLL | PB | 46,XX,add(4)(q35),del(13)(q12q22) [2]/ 46,XX[19]  nuc ish(D13S319x1, LAMP1x2) [70/211],(MYBx2)[211], (ATM,TP53)x2 [208],(D12Z3x2)[211], (IGHx2)[233] | CGH | 13 | q14.2 - q14.3 | 50,588,799 | 51,625,488 | 1,036,689 | Loss | Mosaic | 15 | *DLEU2, DLEU1, DLEU7* |
| CP-22 | F/68 | CLL | PB | 46,XX[18].nuc ish(D13S319x1, LAMP1x2)[38/233],(D13S219x0, LAMP1x2,D12Z3x2)[95/233], (MYBx2)[217],(ATM,TP53)x2[209],(IGHx2) [274] | CGH | 13 | q13.3 - q21.2 | 37,776,424 | 60,597,522 | 22,821,098 | Loss | Mosaic | 238 | *RB1, DLEU2, DLEU1* |
|  |  |  |  |  |  | 13 | q14.2 - q14.3 | 49,046,742 | 51,729,918 | 2,683,176 | Loss hmz | Mosaic | 40 | *RB1, DLEU2, DLEU1, DLEU7* |
| CP-23 | M/61 | CLL | LN | 56,XY,+X,+1,+4,+6,i(6)(p10),+7,+8,+9, der(13;21)(p10;q10),+der(13;21) (p10;q10)x2,hsr(14)(p11.2),+19,+20, +21,hsr(22)(p11.2)[cp15]  **Previous BM:** 46,XY,del(13)(q12q22)[2]/46,idem, add(3)(q27),del(6)(q21q25), del(14)(q11.2q22)[5]/46,XY[14]  **Previous BM:** 46,XY,t(1;3)(p13;p25),del(5)(q22q33), t(6;14)(p25;q32),del(11)(q22q23),-13, -18, -20,+mar[cp3]/46,XY[5]  **Previous PB**: 46,XY,t(1;3)(p13;p25),t(6;14)(p25; q32),-13, -18,-20,+mar[14]/46,XY[6]  **Previous LN:** 46,XY,del(13)(q12q22)[1]/44~46,XY, t(1;3)(p13;p25),del(5)(q22q33),del(6) (q21q25),t(6;14)(p25;q32), del(11)(q22q23), -18,-20,+mar[cp5]/46,XY[10] | CGH+SNP | 1 | p36.33 - q44 | 564,424 | 249,197,762 | 248,633,339 | Gain | Trisomy 1 | 3,398 |  |
|  |  |  |  |  |  | 2 | p25.3 - p23.1 | 42,444 | 30,157,520 | 30,115,077 | Gain | additional findings Tetrasomy 2p | 269 |  |
|  |  |  |  |  |  | 2 | p23.1 - q11.1 | 30,157,520 | 96,236,712 | 66,079,193 | Gain | additional findings  Trisomy 2p | 696 |  |
|  |  |  |  |  |  | 2 | p23.1 - p11.2 | 30,873,188 | 89,126,216 | 58,253,029 | LOH | additional findings | 564 | *FANCL* |
|  |  |  |  |  |  | 2 | q11.1 - q37.3 | 95,665,733 | 237,734,159 | 142,068,427 | LOH | additional findings | 1,373 | *ZAP70* |
|  |  |  |  |  |  | 3 | p26.3 - q29 | 256,561 | 197,802,470 | 197,545,910 | LOH | additional findings | 1,939 | *FANCD2* |
|  |  |  |  |  |  | 4 | p16.3 - q35.2 | 12,440 | 190,925,351 | 190,912,912 | Gain | Trisomy 4 | 1,458 |  |
|  |  |  |  |  |  | 6 | p25.3 - p11.1 | 219,055 | 58,713,074 | 58,494,020 | Gain | Isochromosome 6p | 1,037 |  |
|  |  |  |  |  |  | 6 | q11.1 - q27 | 62,789,729 | 170,862,336 | 108,072,608 | LOH | additional findings | 855 |  |
|  |  |  |  |  |  | 7 | p22.3 - q36.3 | 90,167 | 159,118,566 | 159,028,400 | Gain | Trisomy 7 | 1,850 |  |
|  |  |  |  |  |  | 8 | p23.3 - q24.3 | 73,810 | 146,231,971 | 146,158,162 | Gain | Trisomy 8 | 1,320 |  |
|  |  |  |  |  |  | 8 | q24.21 | 128,711,393 | 129,612,385 | 900,993 | Amp | additional findings | 8 | *MYC, PVT1* |
|  |  |  |  |  |  | 9 | p24.3 - q34.3 | 163,161 | 141,122,114 | 140,958,954 | Gain | additional findings Tetrasomy 9 | 1,504 |  |
|  |  |  |  |  |  | 11 | p15.4 - p11.12 | 7,591,627 | 49,111,467 | 41,519,841 | LOH | additional findings | 421 | *WT1* |
|  |  |  |  |  |  | 13 | q11 - q34 | 19,227,564 | 115,107,245 | 95,879,682 | Gain | Trisomy 13 | 868 |  |
|  |  |  |  |  |  | 13 | q12.11 - q34 | 19,584,477 | 113,286,248 | 93,701,772 | LOH | additional findings | 814 | *FLT3, RB1, BRCA2* |
|  |  |  |  |  |  | 17 | p13.3 - q25.3 | 72,083 | 80,974,162 | 80,902,080 | LOH | additional findings | 1,753 | *TP53, NF1, BRCA1* |
|  |  |  |  |  |  | 19 | p13.3 - q13.43 | 277,373 | 59,085,576 | 58,808,204 | Gain | Trisomy 19 | 2,030 |  |
|  |  |  |  |  |  | 19 | q11 - q13.43 | 28,015,823 | 59,085,576 | 31,069,754 | Gain | additional findings Tetrasomy 19q | 1,172 |  |
|  |  |  |  |  |  | 20 | p13 - q13.33 | 80,198 | 62,889,434 | 62,809,237 | Gain | Trisomy 20 | 893 |  |
|  |  |  |  |  |  | 21 | q11.2 - q22.3 | 14,420,615 | 48,080,926 | 33,660,312 | Gain | additional findings Tetrasomy 21 | 444 |  |
|  |  |  |  |  |  | 22 | q11.1 - q13.33 | 17,450,515 | 51,103,692 | 33,653,178 | LOH | additional findings | 806 | *MN1, NF2* |
|  |  |  |  |  |  | X | p22.33 - q28 | 175,295 | 154,908,471 | 154,733,177 | Gain | Disomy X | 1,682 |  |
| **Sample** | **Gender/Age, yrs** | **Dx** | **Specimen type** | **Karyotype and FISH results** | **Microarray** | **Chr** | **Cytoband** | **Start** | **Stop** | **Size, bp** | **Copy number** | **Comments** | **Total Genes** | **Critical Genes/regions** |
| CP-24 | M/65 | PCM | BM | 57,XY,+3,+4,+5,+7,-8,+9,+11, +15,+15,+18,+19, +21,+21[4]/46,XY[16]  nuc ish(IGHx2,CCND1x3)[147/257], (D17Z1x1,TP53x2)[15/263],(D13S319,  LAMP1)x2[225],(D5S23,D5S721)x3 [144/254],(D7Z1x3)[131/254], (ASS1x3)[140/254] | CGH | 3 | p26.3 - q29 | 68,644 | 197,840,323 | 197,771,679 | Gain | Mosaic trisomy 3 | 1,941 |  |
|  |  |  |  |  |  | 4 | p16.3 - p13 | 10,199 | 43,406,397 | 43,396,198 | Gain | Mosaic trisomy 4p | 384 |  |
|  |  |  |  |  |  | 4 | p13 - q35.2 | 43,406,397 | 191,043,602 | 147,637,205 | Gain | Mosaic trisomy 4 | 1,088 |  |
|  |  |  |  |  |  | 5 | p15.33 - q35.3 | 22,149 | 180,712,263 | 180,690,114 | Gain | Mosaic trisomy 5 | 1,613 |  |
|  |  |  |  |  |  | 7 | p22.3 - q36.3 | 65,558 | 159,125,676 | 159,060,118 | Gain | Mosaic trisomy 7 | 1,850 |  |
|  |  |  |  |  |  | 8 | p23.3 - p11.21 | 176,717 | 42,901,851 | 42,725,134 | Loss | Mosaic monosomy 8 | 500 |  |
|  |  |  |  |  |  | 9 | p24.3 - q34.3 | 163,131 | 141,122,114 | 140,958,983 | Gain | Mosaic trisomy 9 | 1,504 |  |
|  |  |  |  |  |  | 11 | p15.5 - q25 | 217,046 | 134,934,196 | 134,717,150 | Gain | Mosaic trisomy 11 | 2,137 |  |
|  |  |  |  |  |  | 15 | q11.1 - q26.3 | 20,063,386 | 102,480,888 | 82,417,502 | Gain | Mosaic tetrasomy 15 | 1,214 |  |
|  |  |  |  |  |  | 18 | p11.32 - q23 | 69,754 | 78,010,032 | 77,940,278 | Gain | Mosaic trisomy 18 | 569 |  |
|  |  |  |  |  |  | 19 | p13.3 - q13.43 | 266,117 | 59,085,576 | 58,819,459 | Gain | Mosaic trisomy 19 | 2,030 |  |
|  |  |  |  |  |  | 21 | p11.2 - q22.3 | 9,412,632 | 48,080,926 | 38,668,294 | Gain | Mosaic tetrasomy 21 | 464 |  |
| CP-25 | F/88 | PCM | BM | 45,X,-X[3]/46,XX[17].nuc ish(IGHx2)[202],(IGHx2,CCND1x2~3) [18/244],(TP53,D17Z1)x2[202], (D13S319,LAMP1)x2[243],(D5S23, D5S721x3)[23/340],(D7Z1,ASS1)x2 [340],(IGH,MAF)x2[278],(IGH, FGFR3)x2[236] | CGH | 3 | p26.3 - q29 | 68,644 | 197,840,323 | 197,771,679 | Gain | additional findings Mosaic trisomy 3 | 1,941 |  |
|  |  |  |  |  |  | 5 | p15.33 - q35.3 | 22,149 | 180,712,263 | 180,690,114 | Gain | Mosaic trisomy 5 | 1,613 |  |
|  |  |  |  |  |  | 9 | p24.3 - q34.3 | 163,131 | 141,122,114 | 140,958,983 | Gain | additional findings Mosaic trisomy 9 | 1,504 |  |
|  |  |  |  |  |  | 11 | p15.5 - q25 | 217,046 | 134,934,196 | 134,717,150 | Gain | additional findings Mosaic trisomy 11 | 2,137 |  |
|  |  |  |  |  |  | 15 | q11.1 - q26.3 | 20,063,386 | 102,480,888 | 82,417,502 | Gain | additional findings Mosaic trisomy 15 | 1,214 |  |
|  |  |  |  |  |  | 19 | p13.3 - q13.43 | 266,117 | 59,085,576 | 58,819,459 | Gain | additional findings Mosaic trisomy 19 | 2,030 |  |
|  |  |  |  |  |  | 21 | p11.2 - q22.3 | 9,412,632 | 48,080,926 | 38,668,294 | Gain | additional findings Mosaic trisomy 21 | 464 |  |
|  |  |  |  |  |  | X | p22.33 - q28 | 175,295 | 154,908,471 | 154,733,177 | Loss | Monosomy X | 1,682 |  |
| **Sample** | **Gender/Age, yrs** | **Dx** | **Specimen type** | **Karyotype and FISH results** | **Microarray** | **Chr** | **Cytoband** | **Start** | **Stop** | **Size, bp** | **Copy number** | **Comments** | **Total Genes** | **Critical Genes/regions** |
| K-1 | 7/M | AML | Cell line | 46~47,X,-Y,add(2)(q33),+der(4) (p12→q13)x2,add(7)(p12),t(8;21) (q22;q22),der(9)t(9;15)(p21;q21),+10, der(12)t(9;12)(q13;p12),-13,del(15) (q11.2),der(16)t(13;16)(q14;q12-13), add(17)(p12)[cp3]/47~49,sl, +der(4)(p12→q13),add(5)(p12),+i(5) (p10)[cp9]/83~95,slx2[cp3] | CGH | 2 | q21.2 - q33.2 | 134,912,510 | 204,336,695 | 69,424,185 | Gain | additional findings | 552 |  |
|  |  |  |  |  |  | 2 | q33.2 - q37.3 | 204,360,302 | 243,040,276 | 38,679,974 | Loss | der(2)add(2q) | 460 |  |
|  |  |  |  |  |  | 3 | q13.2 - q13.31 | 112,945,433 | 114,189,766 | 1,244,333 | Gain | additional findings | 27 |  |
|  |  |  |  |  |  | 4 | p12 - q13.1 | 46,445,453 | 61,576,425 | 15,130,972 | Gain | segmental tetrasomy 4 | 94 |  |
|  |  |  |  |  |  | 4 | q11 - q12 | 52,697,788 | 59,369,224 | 6,671,436 | Amp | additional findings | 66 | *PDGFRA, KIT* |
|  |  |  |  |  |  | 5 | p15.33 - p11 | 22,149 | 46,350,808 | 46,328,659 | Gain | i(5p) | 314 |  |
|  |  |  |  |  |  | 7 | p22.3 - p15.3 | 65,558 | 21,013,391 | 20,947,833 | Loss | der(7)add(7p) | 195 |  |
|  |  |  |  |  |  | 7 | p15.3 - p11.2 | 21,037,997 | 56,854,691 | 35,816,694 | Gain | additional findings | 409 | *EGFR* |
|  |  |  |  |  |  | 8 | q21.3 | 93,048,668 | 93,066,592 | 17,924 | Loss | additional findings t(8;21) breakpoint | 1 | *RUNX1T1* |
|  |  |  |  |  |  | 8 | q24.13 - q24.3 | 123,702,191 | 146,293,435 | 22,591,244 | Gain | additional findings | 239 |  |
|  |  |  |  |  |  | 9 | p24.3 - p21.1 | 163,131 | 29,299,751 | 29,136,620 | Loss | der(9)t(9;15) | 213 | *JAK2, CDKN2A, CDKN2B* |
|  |  |  |  |  |  | 10 | p15.3 - q26.3 | 136,361 | 135,499,049 | 135,362,689 | Gain | Trisomy 10 | 1,396 |  |
|  |  |  |  |  |  | 11 | p15.3 | 217,046 | 2,195,440 | 1,978,394 | Gain | additional findings | 87 |  |
|  |  |  |  |  |  | 12 | p13.33 - p12.3 | 162,848 | 16,743,603 | 16,580,755 | Loss | der(12)t(9;12) | 348 | *ETV6* |
|  |  |  |  |  |  | 12 | p12.3 | 16,763,349 | 18,881,703 | 2,118,354 | Gain | additional findings | 12 |  |
|  |  |  |  |  |  | 13 | q11 - q14.11 | 19,066,577 | 41,579,497 | 22,512,920 | Loss | der(16)t(13;16) | 308 | *FLT3, BRCA2* |
|  |  |  |  |  |  | 14 | q21.3 | 50,122,371 | 50,219,072 | 96,701 | Loss | additional findings | 3 |  |
|  |  |  |  |  |  | 15 | q11.1 - q21.1 | 20,063,386 | 46,661,967 | 26,598,581 | Loss | segmental monosomy 15 | 502 |  |
|  |  |  |  |  |  | 16 | p13.3 - p11.2 | 100,293 | 31,689,802 | 31,589,509 | Loss | segmental monosomy 16p | 632 | *PALB2* (*FANCN*) |
|  |  |  |  |  |  | 16 | p11.2 | 31,721,084 | 31,959,133 | 238,049 | Gain | additional findings | 8 |  |
|  |  |  |  |  |  | 16 | q11.2 - q12.1 | 46,965,235 | 47,462,718 | 497,483 | Amp | additional findings | 5 |  |
|  |  |  |  |  |  | 17 | p13.3 - p11.2 | 11,807 | 22,226,321 | 22,214,515 | Loss | additional findings | 543 | TP53 |
|  |  |  |  |  |  | 17 | q21.33 - q22 | 48,277,936 | 52,059,263 | 3,781,327 | Gain | additional findings | 38 |  |
|  |  |  |  |  |  | 17 | q22 - q25.3 | 56,779,604 | 81,124,227 | 24,344,623 | Gain | additional findings | 454 |  |
|  |  |  |  |  |  | Y | p11.32 - q11.23 | 13,303 | 59,031480 | 59,018,178 | Loss | -Y | 53 |  |
| **Sample** | **Gender/Age, yrs** | **Dx** | **Specimen type** | **Karyotype and FISH results** | **Microarray** | **Chr** | **Cytoband** | **Start** | **Stop** | **Size, bp** | **Copy number** | **Comments** | **Total Genes** | **Critical Genes/regions** |
| K-3 | 57/M | AML | Cell line | 46,XY,t(2;5)(p13;q33),t(3;7)(q26;q22), del(5)(q15),-8,del(9)(q32), add(12)(p11),add(16)(q13),+mar[20] | CGH | 2 | p22.2 - p22.1 | 38,548,713 | 40,304,903 | 1,756,190 | Loss | additional findings | 22 |  |
|  |  |  |  |  |  | 2 | p22.1 | 41,021,766 | 41,478,035 | 456,269 | Loss | additional findings | 0 |  |
|  |  |  |  |  |  | 2 | p16.3 | 50,538,743 | 51,929,811 | 1,391,068 | Loss | additional findings | 2 |  |
|  |  |  |  |  |  | 3 | p21.31 | 46,987,723 | 49,031,490 | 2,043,767 | Loss | additional findings | 53 |  |
|  |  |  |  |  |  | 3 | q26.2 | 168,932,905 | 169,041,367 | 108,462 | Loss | additional findings t(3;7) breakpoint | 1 | *MECOM (EVI1)* |
|  |  |  |  |  |  | 4 | q13.1 | 60,180,130 | 61,151,359 | 971,229 | Loss | additional findings | 0 |  |
|  |  |  |  |  |  | 5 | q14.2 - q33.3 | 81,992,149 | 156,432,861 | 74,440,712 | Loss | del(5) | 694 | 5q (*EGR1*) deletion |
|  |  |  |  |  |  | 5 | q34 - q35.3 | 160,354,523 | 180,712,263 | 20,357,740 | Loss | del(5) | 244 | *NPM1* |
|  |  |  |  |  |  | 7 | p22.3 - p15.2 | 2,397,553 | 26,497,940 | 24,100,387 | Loss | additional findings | 210 |  |
|  |  |  |  |  |  | 7 | q22.1 | 99,039,777 | 99,121,688 | 81,911 | Loss | additional findings t(3;7) breakpoint | 8 |  |
|  |  |  |  |  |  | 7 | q34 - q36.1 | 142,497,020 | 149,062,776 | 6,565,756 | Loss | additional findings | 105 | 7q (*EZH2*) deletion |
|  |  |  |  |  |  | 7 | q36.3 | 155,870,347 | 156,353,975 | 483,628 | Loss | additional findings | 4 |  |
|  |  |  |  |  |  | 9 | q21.33 | 87,682,757 | 88,198,392 | 515,635 | Loss | additional findings | 3 |  |
|  |  |  |  |  |  | 9 | q32 - q33.2 | 115,122,960 | 123,820,317 | 8,697,357 | Loss | del(9) | 70 |  |
|  |  |  |  |  |  | 10 | q21.3 - q26.3 | 66,251,849 | 135,499,049 | 69,247,200 | Gain | additional findings | 806 | *PTEN* |
|  |  |  |  |  |  | 12 | p13.2 - p11.23 | 11,646,474 | 27,093,200 | 15,446,726 | Loss | der(12)/add(12) | 146 | *ETV6, KRAS* |
|  |  |  |  |  |  | 20 | q12 - q13.2 | 40,186,203 | 50,653,152 | 10,466,949 | Loss | additional findings | 178 | 20q deletion |
| Dx-diagnosis, AML - acute myeloid leukemia, MDS - myelodysplastic syndrome, B-ALL - B-cell acute lymphocytic leukemia, CLL- chronic lymphocytic leukemia, CML - chronic myeloid leukemia, PCM - plasma cell myeloma, MPN - myeloproliferative neoplasm, PV - polycythemia vera. | | | | | | | | | | | | | | |
